# A spatially discretized convolutional neural mass model for studying meso-scale spatio-temporal transformations in the rat hippocampus

**DOI:** 10.1007/s10827-026-00950-9

**Published:** 2026-08-10

**Authors:** Duy-Tan J. Pham, Gene J. Yu, Gianluca Lazzi, Jean-Marie C. Bouteiller

**Affiliations:** 1 Neural Systems Computational Modeling Lab (NESCOM), University of Southern California, Los Angeles, CA USA; 2 Center for Neural Engineering (CNE), University of Southern California, Los Angeles, CA USA; 3 Brain Stimulation Engineering Lab, Department of Psychiatry and Behavioral Sciences, Duke University, Durham, NC USA; 4 Alfred E. Mann Department of Biomedical Engineering, Viterbi School of Engineering, University of Southern California, Los Angeles, CA USA; 5 Institute for Technology and Medical Systems (ITEMS), Keck School of Medicine, University of Southern California, Los Angeles, CA USA; 6 Department of Electrical Engineering, Viterbi School of Engineering, University of Southern California, Los Angeles, CA USA

**Keywords:** Neural mass models, Multi-scale modeling, Large-scale models, Input-output modeling, Forward modeling, Volterra series, Meso-scale, Hippocampus

## Abstract

The brain operates across multiple spatial and temporal scales, necessitating computationally efficient models that link micro-scale mechanisms to meso- and macro-scale dynamics. Here, we introduce a novel convolutional neural mass model (CNMM) that computes the meso-scale activity of spatially discretized neural populations (“neural masses") in the rat hippocampal CA3 subregion. The CNMM employs a kernel-based architecture, leveraging first-order Volterra expansions with Laguerre (temporal) and Chebyshev (spatial) basis functions to transform input spike densities from entorhinal cortex (EC), dentate gyrus (DG), and neighboring CA3 masses into output CA3 spike density. The model was trained and validated using data from a biophysically detailed large-scale mechanistic model (LSM) simulating exploratory behavior. The CNMM achieved high predictive accuracy for spike density across 32 neural masses spanning the entire extent of CA3 (mean correlation coefficient *R = 0.951 ± 0.027*) and replicated theta and beta oscillations consistent with experimental findings. When extended for forward modeling, the CNMM accurately predicted local field potentials (LFPs) at a single neural mass (*R = 0.952*), demonstrating feasibility of this approach. Kernel analysis revealed topographic gradients in afferent integration, with DG inputs dominating proximally (CA3c) and associational connections distally (CA3a), aligning with anatomical gradients. Compared to the LSM, the CNMM provided a 658-fold speedup in simulation time, 322-fold reduction in memory usage, and 183-fold less disk space for LFP predictions. This framework offers a scalable, efficient approach for meso-scale modeling of neural tissue, bridging detailed simulations with empirical data and laying groundwork for future investigations into both normal and pathological brain function.

**Supplementary Information:**

The online version contains supplementary material available at https://doi.org/10.1007/s10827-026-00950-9.

## Introduction

The brain is inherently a multi-scale system, operating across many spatial and temporal scales that interact to produce cognition and behavior. Spatially, it spans from the molecular interactions within ion channels (nanometers), cellular dynamics of individual neurons (micrometers), to macro-scale networks of entire brain regions (millimeters/centimeters). Temporally, brain dynamics unfold over multiple timescales, ranging from vesicular release of neurotransmitter (microseconds), to firing of action potentials (milliseconds), to synaptic plasticity and learning (seconds to hours), to neurodegenerative processes (years). Drug-based medical treatments constitute a clear illustration of these multi-scale interactions, where molecular-level changes lead to alterations in behavior. Computational neuroscience has been strongly driven by efforts to understand how drugs work and the mechanisms behind neural disorders, leading to the creation of a wealth of sophisticated, biophysically realistic models often siloed in their respective scale(s). Linking these models would enable a multi-scale approach to accurately propagate the functional outcomes from lower levels to higher levels, forming a comprehensive and holistic representation of the multi-scale mechanisms that underlie a therapeutic or degenerative condition. However, simply combining these detailed models at different scales requires a prohibitive amount of computational resources when modeling at the large-scale.

Large-scale models of neural systems involve simulating the activity of neuronal networks containing thousands to millions of neurons to capture the interactions and dynamics of neural populations of entire brain regions or subregions. Note, throughout the manuscript, the term “large-scale" refers to the size of the model (i.e., a large number of neurons being simulated), whereas references to micro-, meso-, macro-, or multi-scale refer to different spatial extents in the brain. These models represent the activity taking place at the meso-scale and are important for understanding differences in processing or topographic variations in processing that occur within a region. Accurately representing the dynamics at the meso-scale is crucial as it bridges the micro-scale (subcellular and neuron levels) to the macro-scale (inter-regional and brain levels). Izhikevich and Edelman developed a large-scale thalamocortical model containing 1 million neurons capable of reproducing neuronal responses found *in vitro* in rats by using a simplified neuron model that is represented by coupled differential equations (Izhikevich & Edelman, 2008). In 2015, colleagues from the Blue Brain Project developed a comprehensive large-scale model of the microcircuitry within the neocortex which was able to reproduce *in vitro* and *in vivo* experiments as well as emergent properties found in the brain (Markram et al., 2015). Biologically informed large-scale mechanistic models have also been developed to study the spatio-temporal encodings within the rat hippocampus which incorporate anatomically-constrained connectivity, conductance based synaptic dynamics, realistic neuron morphologies, multi-compartment circuits for representing neurons, topography, subcellular mechanisms (e.g., multiple ion channels), over a million neurons, and more. Hendrickson et al. (2016); Yu et al. (2013, 2018). Such detailed and high-dimensional models come with a heavy computational burden that require simulations to take place on either supercomputers or high-performance computer clusters thus motivating the necessity for more efficient methodologies that reduce computational demand.

Significant reduction of dimensionality at the meso-scale is achieved with neural mass models (NMMs) in which the dynamics of neuronal populations is quantified by their mean activity (Breakspear, 2017). Some of the earliest examples of NMMs were from Wilson and Cowan (1972); Jansen and Rit (1995) in which the firing rates of neural populations—excitatory and inhibitory—were modeled using coupled differential equations which continued to become key components in the Virtual Brain modeling platform (Sanzleon et al., 2013). This framework was extended in Gerstner (1995) and Brunel (2000) in which mean-field models were used to derive population dynamics from biophysical principles of spiking neurons, enabling analysis of network stability and irregular firing regimes. However, these models often abstract away input-specific transformations, focusing instead on averaged excitatory-inhibitory interactions without explicit handling of topographic or afferent-specific variations. Because NMMs represent aggregate activity of entire populations, they are natural contenders for addressing the forward problem, which entails predicting observable signals (e.g., electromagnetic fields or hemodynamic responses) generated by underlying neural sources. Consequently, NMMs are widely employed in forward modeling to simulate macroscopic signals such as local field potentials (LFPs), electroencephalography (EEG), magnetoencephalography (MEG), and functional magnetic resonance imaging/blood oxygenation level dependent (fMRI/BOLD) signals (Bojak et al., 2010, 2011; Telenczuk et al., 2020; Tesler et al., 2022). This has led to clinical applications involving simulation of brain oscillations, giving insight into the mechanisms underlying different brain states and neuropathologies. For example, a neural mass modeling approach was employed to simulate movement-related beta decrease (MRBD) and post-movement beta rebound (PMBR) in motor cortex, elucidating the reduced PMBR observed in schizophrenia patients (Byrne et al., 2017). In Song et al. (2020), a NMM methodology was used for early epileptic seizure detection through analysis of EEG data. In contrast to the forward problem, the inverse problem involves inferring the neural sources that give rise to observed signals. In Li et al. (2021), a neural mass modeling framework coupled with a hemodynamic model was utilized to infer mesoscopic parameters and effective connectivities from empirical macroscopic fMRI BOLD signals. This inversion technique led to the identification of the neural circuits most likely involved in major depressive disorder. Furthermore, a multi-scale mean-field model of the CA1 region of the hippocampus was developed in Tesler et al. (2024) in which meso-scale dynamics were replicated such as the theta and gamma rhythms. Building on this methodology, a very similar approach was applied to evaluating the impact of molecular mechanisms, such as the anesthetic effects on synaptic receptors, on whole-brain dynamics (Sacha et al., 2025). Although granularity at the individual neuron level is lost by aggregating activity across populations, NMMs enable the capture of population-level dynamics that encode complementary information not fully represented in single-unit spiking. For example, multi-scale decoding frameworks have demonstrated that local field potentials (LFPs)—reflecting synchronized population activity—provide synergistic improvements in behavioral decoding when combined with spike data, suggesting that information is encoded at multiple spatio-temporal scales (Hsieh et al., 2019).

Input-output modeling refers to a class of data-driven and statistical approaches that aim to directly map inputs to outputs mathematically in contrast to mechanistic approaches that aim to model the underlying biology of a system. Because input-output models circumvent the complexity of mechanistic approaches, they achieve computational efficiency and require no prior knowledge of the system. However, they are limited in that they provide less mechanistic insight into the system. Some examples of input-output modeling techniques include generalized linear models (GLMs), deep neural networks, and the Volterra functional power series. For instance, Pillow et al. (2008) employed a GLM to predict spike responses of a population of retinal ganglion cells by applying spatio-temporal filters on visual stimuli and coupling filters between neurons. Modeling via the Volterra series involves convolutions between inputs and higher order kernels within a memory window to predict the target output and can represent nonlinear dynamics. In Friston et al. (2000), the Volterra framework was used to validate the Balloon-Windkessel model, demonstrating that it could capture the nonlinearity associated with the BOLD signal and neuronal activity. Expansion of Volterra kernels using Laguerre basis functions were shown to be an effective, yet computationally light methodology in simulating physiological signals and gave rise to a rich family of Laguerre-Volterra models spanning the many different scales of the brain (Marmarelis, 1993). For example, dynamics of the membrane potential of *in vitro* CA3 pyramidal cells were captured using the Laguerre-Volterra framework, enabling identification of kernel differences between control and epileptogenic conditions (Kang et al., 2010). Furthermore, the Laguerre-Volterra approach was expanded and formed the computational basis for modeling CA1 pyramidal cell spiking, and ultimately, a hippocampal prosthetic for restoring memory function (Berger et al., 2011; Song et al., 2009, 2018). In Kostoglou et al. (2012, 2024), the Laguerre-Volterra methodology was used to predict spike times in the subthalamic nucleus from input LFP signals and provided a data-driven protocol for optimal electrode placement in treating Parkinson’s disease using deep brain stimulation. Additionally, nonlinear synaptic dynamics were accurately represented using Laguerre-Volterra expansion, resulting in computationally efficient modeling of glutamatergic receptors and enabling large-scale multi-scale models of neural systems (Hu et al., 2015, 2018).

In this paper, we propose a novel kernel-based, convolutional neural mass model (CNMM) to represent the mean activity of spatially discretized bins (“neural masses") of the CA3 subregion within the rat hippocampus. The model is trained and validated using meso-scale data generated from an anatomically detailed large-scale mechanistic model of the rat hippocampus. The architecture of the CNMM is composed of Volterra kernels, expanded using Laguerre and Chebyshev basis functions. We demonstrate that our model can successfully capture the meso-scale dynamics across the CA3 topography and illustrate its utility in forward modeling by accurately predicting local field potential signals. We also show the significant reduction in computational demands compared to that of the large-scale mechanistic model. Finally, we discuss the implications of this work and how it serves as a foundational proof-of-concept for future applications in computational neuroscience.

## Methods

### Spatially discretized neural masses across CA3 extent

**Fig. 1:**
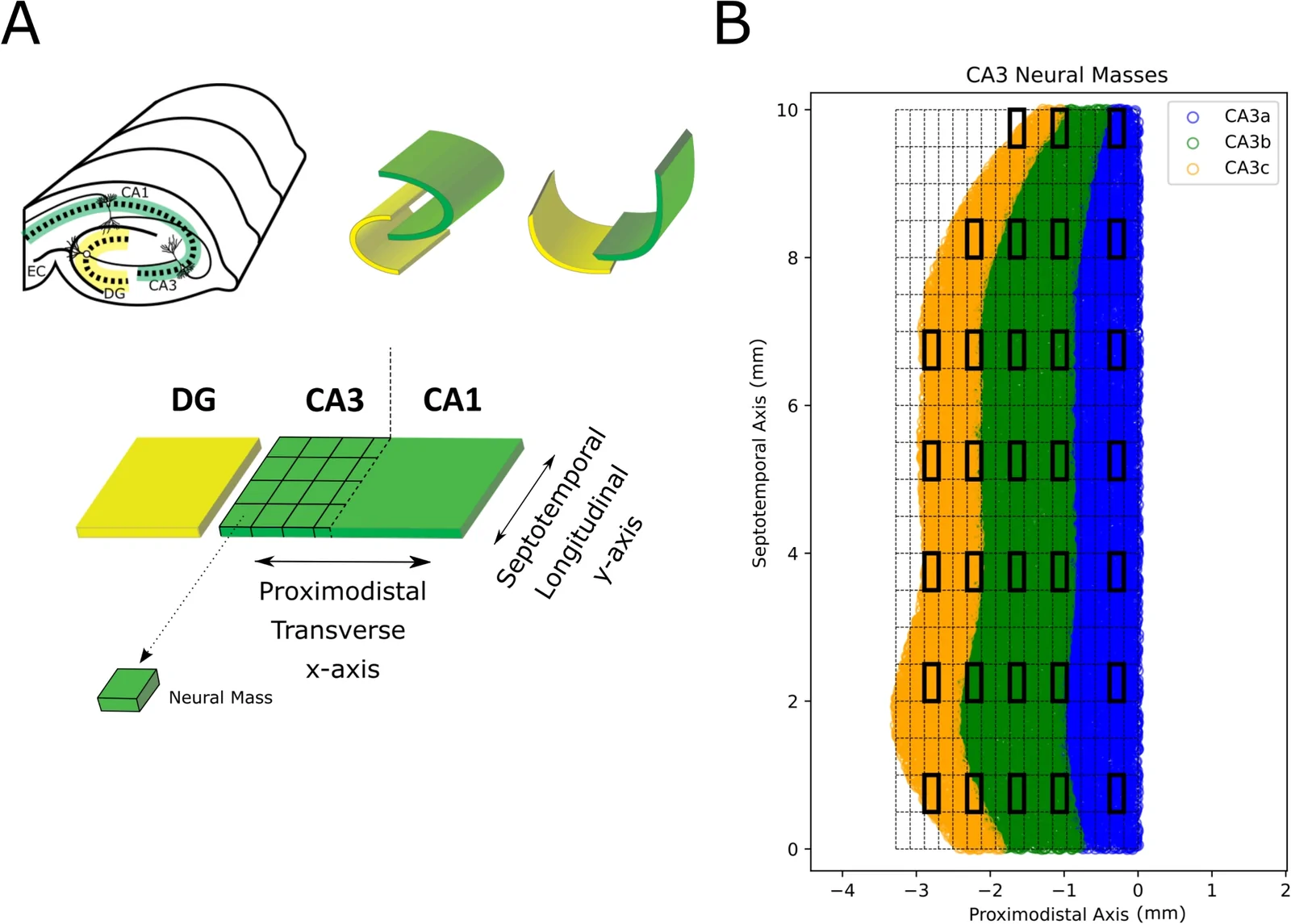
Discrete CA3 neural masses. (**A**) The hippocampus was flattened to create a standard coordinate system. The CA3 subregion was divided into discrete spatial bins, or neural masses. (**B**) The precise map of CA3 neural masses used in this study are shown. The major axes include the proximodistal axis and the septotemporal axis (units of mm). A proximodistal position of zero represents the interface between CA3 and CA1. Neural masses were 500 *μ *m by 200 *μ *m. Thick black rectangles indicate neural masses that were optimized in this study. The three subfields (CA3a, CA3b, and CA3c) are shown and color coded

The CA3 subregion was selected as the focus of this study for several reasons. CA3 receives diverse external inputs from the entorhinal cortex (via the perforant path), dentate gyrus (via mossy fibers), strong recurrent inputs from other CA3 pyramidal cells via associational connections, and inhibitory input from local basket cells that mediate both feedforward and feedback inhibition. This combination of multiple distinct inputs converging onto a recurrent network creates a rich computational environment in which inputs of differing strength, anatomical termination, and excitatory-inhibitory balance interact with internal circuit dynamics. In particular, CA3 possesses a dense recurrent excitatory network through its associational connections, combined with robust feedback inhibition. In addition, CA3 exhibits well-characterized topographic gradients along both the proximodistal and septotemporal axes in terms of cellular composition, connectivity, and input integration. These features make CA3 particularly well-suited for investigating meso-scale input–output transformations using a spatially discretized, multi-input modeling framework. Furthermore, CA3 is a well-studied region with extensive anatomical and physiological data available, and it is supported by our previously developed large-scale mechanistic model of the rat entorhinal-dentate-CA3 system, enabling training and validation of the CNMM. For this study, a “neural mass" was defined to be a population of neurons that are contained within a spatial bin. A two-dimensional bin was used based on a flattened representation of the hippocampal map in which the two main axes are the transverse/proximodistal axis (x-axis) and the longitudinal/septotemporal axis (y-axis). The CA3 subregion of the rat hippocampus was divided into multiple neural masses of the same bin size, 0.2 mm (proximodistal) by 0.5 mm (septotemporal), resulting in a total of 304 neural masses. On average, a neural mass contained approximately 82 CA3 pyramidal cells and 33 CA3 basket cells in a network representing 1/10th the number of neurons in a true rat hippocampus. To demonstrate that the CNMM can faithfully capture the diverse population dynamics across the CA3 extent, optimization was completed on a subset of 32 neural masses that span the proximodistal and septotemporal axes and includes all three subfields: CA3a, CA3b, CA3c. These 32 neural masses were chosen because they provide broad coverage of the CA3 topography while avoiding redundancy, as neighboring neural masses tend to produce similar firing patterns. The remaining 272 neural masses are present in the full model but were not individually optimized in this study in order to significantly reduce computational costs. Figure 1 provides visualization of the two-dimensional CA3 neural masses. As these 32 neural masses are distributed across all three CA3 subfields, they are expected to broadly represent the full set of 304 neural masses.

### Data from a large-scale mechanistic model

The CNMM is a mesoscopic input-output analog to our previously developed large-scale mechanistic model, described extensively in Yu et al. (2018, 2019, 2020), of the rat entorhinal-dentate-CA3 system within the hippocampus. Briefly, this large-scale model (hereby, abbreviated as LSM) features neuronal connectivity based on anatomical topography, realistic cell morphologies, conductance-based synaptic dynamics, multi-compartment circuits, topographical gradients within hippocampal subregions, subcellular mechanisms, a large-scale number of neurons, and more. The LSM was implemented using the NEURON simulation environment (Hines & Carnevale, 1997). For this study, the number of neurons contained in the DG and CA3 were reduced to 1/10th of the full-scale number of neurons for simplicity. The neurons involved in this model include:

- 25,000 CA3 pyramidal cells
- 10,000 CA3 basket cells
- 120,000 dentate gyrus granule cells
- 8,086 dentate gyrus basket cells
- 46,000 lateral entorhinal cortex cells
- 66,000 medial entorhinal cortex cells

One of the major inputs to the hippocampus is the entorhinal cortex (layer II), which projects to both the dentate gyrus and CA3 via the perforant path. Grid cells in the medial entorhinal cortex (MEC) encode spatial information through receptive fields that are organized in a hexagonal grid-like pattern (known as the grid field) that collectively span the animal’s spatial environment (Blair et al., 2007; Hafting et al., 2005). Grid field properties vary over the dorsoventral axis of the MEC (Stensola et al., 2012). To capture these physiological details, grid cell firing activity—along with their property gradients—was simulated to provide biologically realistic MEC input to the LSM during an exploration task in an 80 cm by 80 cm square environment for 40 seconds. The trajectory was generated by sampling uniformly distributed random variables including: the speed of the rat (0-30 cm/s), the direction of movement (0$^\circ$-360$^\circ$), and time duration that the rat would move with the sampled velocity (0-500 ms). Movement that would cause the trajectory to exit the square boundaries was reflected so that the path remained inside the boundaries. Every grid cell in the network was uniquely assigned a grid map based on its spatial location within the MEC (Yu et al., 2019). Grid maps were simulated as a summation of three cosine functions, rotated in increments of 60$^\circ$ from each other, to form a hexagonal lattice that projects onto the square environment (Blair et al., 2007).

1 $$\begin{aligned} G({\textbf {r}},\lambda ,\theta ,{\textbf {c}}) = g\left( \sum _{k=1}^3 cos \left( \frac{4\pi }{\sqrt{3\lambda }}z(\theta _k+\theta ) \cdot ({\textbf {r}}-{\textbf {c}}) \right) \right) \end{aligned}$$

2 $$\begin{aligned} z(\theta _k + \theta ) = (cos(\theta _k + \theta ), sin(\theta _k + \theta )) \end{aligned}$$

3 $$\begin{aligned} g(x) = e^{a(x-b)} -1 \end{aligned}$$

where the vector ${\textbf {r}}$ corresponds to the position of the virtual rat at $(x_{rat},y_{rat})$ and the vector ${\textbf {c}} = (x_{offset},y_{offset})$ determines the spatial offset of the grid map. The variable *λ * determines the distance between grid fields. The parameter *a* determines the width of the grid fields while *b = -3/2* sets the minimum value of the function to zero. Grid maps were normalized such that the maximum firing rate of the grid cell was 50 Hz. This peak firing rate value was chosen to ensure adequate drive to the downstream CA3 network while remaining within the range of firing rates observed in some experimental recordings from MEC cells (Prerau et al., 2014). The grid maps define the firing rate of the grid cells as a function of the rat’s position within the square environment. As the rat moves within the environment, the firing rates of the grid cells will change accordingly. An inhomogeneous Poisson renewal process (with firing rates that change as the rat moves through its grid maps) is used to generate spike times for these grid cells. Additional details for implementing grid cell activity are extensively discussed in Yu et al. (2019). Though the MEC contains a variety of functionally distinct cell types, we focused exclusively on grid cells to study spatial exploration in an open environment. Grid cells are the predominant source of periodic spatial information in MEC during navigation and therefore provide the most relevant input for examining how CA3 processes spatially relevant signals. Lateral entorhinal cortical (LEC) neurons provide non-spatial information related to objects and sensation. However, our goal in this study was to only simulate spatial navigation. Therefore LEC neurons were simplified and fired randomly as Poisson point processes with a mean firing rate of 5.50 Hz, contributing noise to the system.

The resulting spiking data generated from the LSM simulation represents behaviorally relevant activity (i.e., exploration) in the rat hippocampus and was used to generate ground truth data which was utilized for training the CNMM. As with traditional neural mass models, the CNMM used a population mean firing rate to describe the dynamics of each neural mass. To estimate the mean firing rate, a spike density function was utilized which converts spike trains into a smooth, continuous, and differentiable signal (Szucs, 1998). To convert the LSM spiking data into ground truth spike density data, the temporal conduction delays were computed for each input cell to a target neural mass by dividing the length of the signal’s path by a propagation velocity (0.27, 0.32 mm/ms for EC, DG, respectively), which represents the latency between the generation of the action potential in the presynaptic neuron and the arrival of the resulting postsynaptic potential at the soma of the target neuron. Using the conduction delays, the input spike time histograms (also referred to as peristimulus time histogram, or PSTH) for all neural masses were calculated. The spike time histogram is a discrete-valued time-varying signal which represents the sum of the binary spiking activity of multiple neurons. At each time bin (width of 2 ms), the number of excitatory input neurons firing (with delay considered) in the LSM that project to a given CA3 neural mass was summed to obtain the input spike time histogram. Similarly, the output spike time histogram was computed by summing the number of CA3 pyramidal cells firing within the neural mass at each time bin. All spike time histograms were standardized by subtracting the signal’s mean and dividing by the signal’s standard deviation to prevent bias in the model stemming from differences in magnitude across inputs. Finally, the standardized spike time histograms were convolved with a Gaussian kernel to produce a smooth spike density function (or simply, "spike density"). This process yielded input spike density traces coming from EC and DG and an output CA3 spike density trace for each CA3 neural mass. The Gaussian kernel is one of the most frequently used kernels for the spike density function and is given by Eq. (4).

4 $$\begin{aligned} k_w(t) = \frac{1}{\sqrt{2\pi } w} \exp \left( \frac{-t^2}{2w^2} \right) \end{aligned}$$

where *w* is an open parameter that defines the bandwidth of the kernel. To pick the optimal value of *w*, the algorithm provided in Shimazaki and Shinomoto (2010) was used, which is spike train dependent. A bandwidth value of 10 ms was chosen for DG and CA3 spike densities while a value of 100 ms was chosen for EC spike densities. Figure A1 visualizes the effect of changing the Gaussian kernel’s bandwidth *w*. In general, increasing the value of *w* will cause the signal to filter out high frequency content and approach a flat, constant signal. For clarity, the Gaussian kernels were only used in the data conversion stage to obtain spike densities and is not to be confused with the kernels that compose the structure of the CNMM.

To summarize, the inputs to the CNMM were the spike densities projected from EC (perforant path), DG granule cells (mossy fiber pathway), and CA3 pyramidal cells (PCs) from neighboring neural masses (associational connections) while the output was the spike density response from CA3 pyramidal cells contained in the target neural mass. The outputs of the CNMM were comprised only of the principal neurons of the subregion, i.e., the pyramidal cells of the CA3. Inhibitory basket cells (BCs) exist in the large-scale mechanistic model within the DG and CA3 subregions, but their contributions to the dynamics in the CNMM were only implicitly represented by the kernels extracted from the methodology. In addition to spike density, the transmembrane currents of each cell in CA3 were recorded. These currents were used to estimate the LFP response, as measured in CA3, and also served as ground-truth data for the CNMM when predicting LFP. More details on LFP estimation are discussed in Section 2.4.

**Fig. 2:**
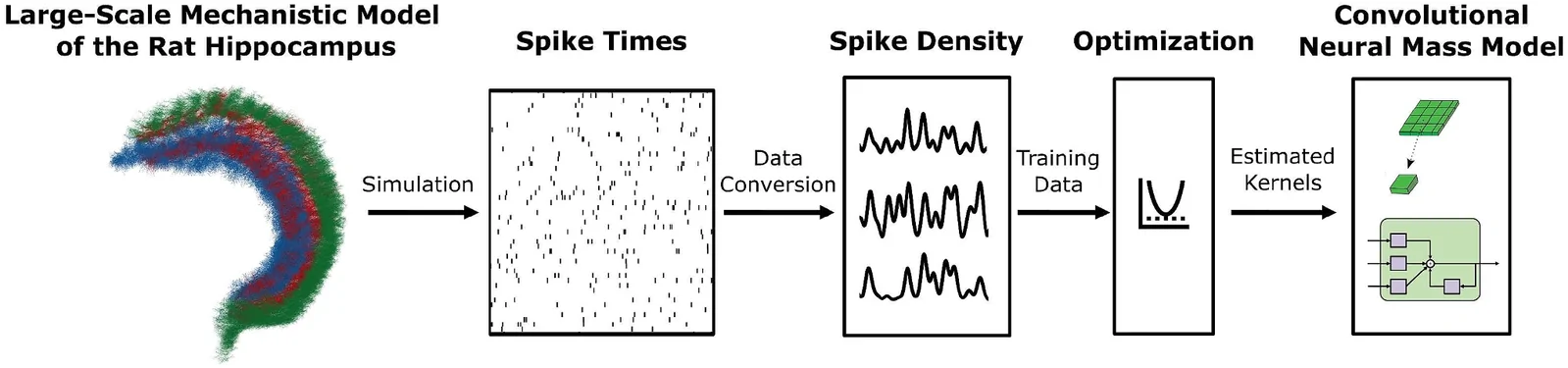
**LSM to CNMM workflow.** A large-scale mechanistic model of the rat hippocampus was simulated, representing rat exploration in an 80 cm by 80 cm square space. The resulting spike times were then converted into input and output spike density traces for every neural mass, providing training data for the convolutional neural mass model. Optimization was performed, resulting in the estimated Volterra kernels that comprised the CNMM

Finally, the high-level workflow from the large-scale mechanistic model of the rat hippocampus to the proposed convolutional neural mass model is illustrated in Fig. 2.

### Architecture of the CNMM

**Fig. 3:**
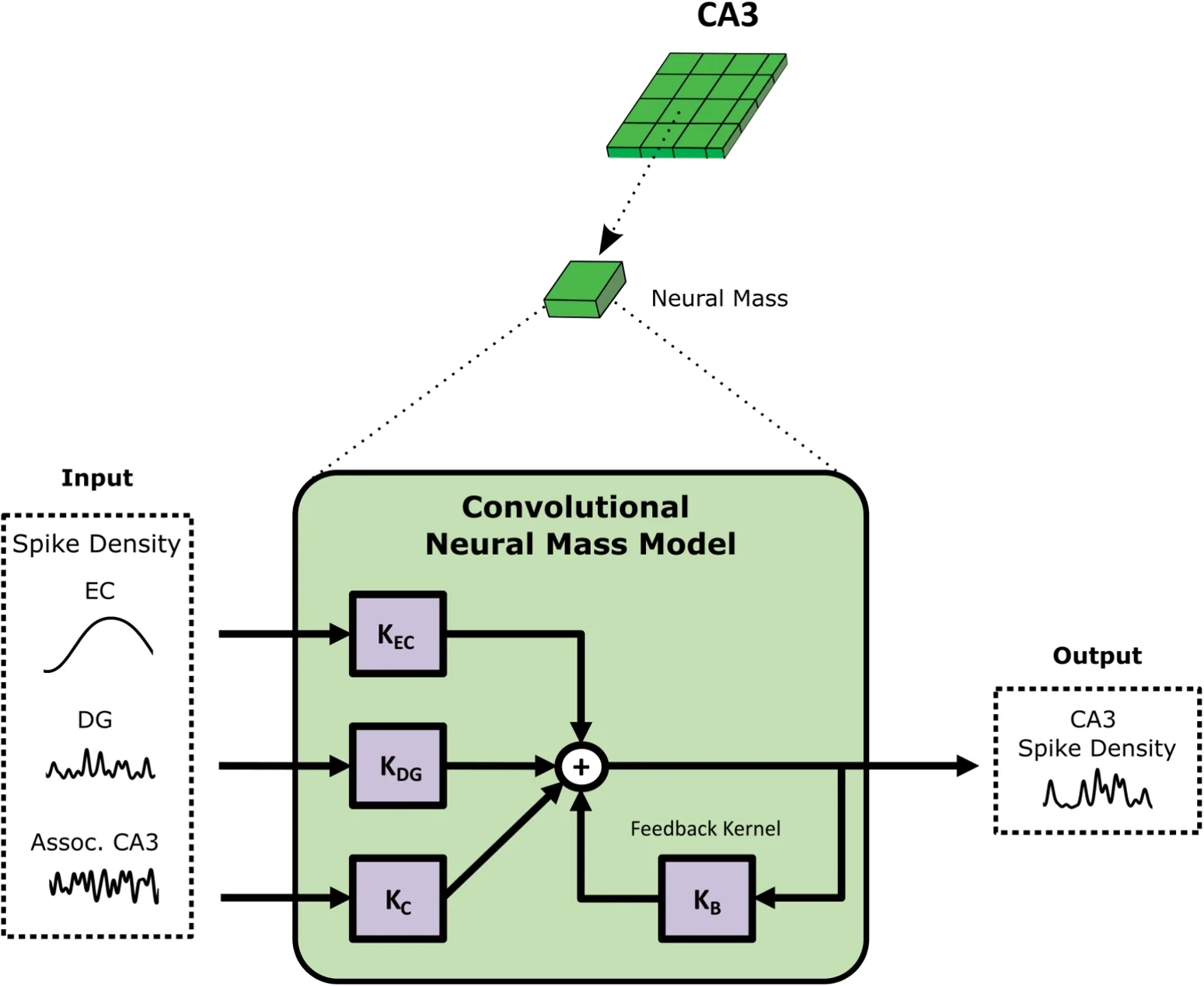
CNMM structure for spike density prediction. The architecture of the SD-CNMM is illustrated. A single CA3 neural mass was represented as a collection of kernels. Inputs to the model include spike densities coming from EC (perforant path), DG (mossy fibers), and neighboring CA3 neural masses (associational connections). Transformation of inputs was performed via convolution with corresponding feedforward kernels. Feedforward kernels were composed of first-order Volterra expansions using a linear combination of basis functions. For EC and DG, the kernels were temporal and were expanded using Laguerre basis functions. The coupling kernel, $K_C$, was a three-dimensional spatio-temporal filter representing the transformation of CA3 associational connections. It was expanded using both Laguerre (temporal) and Chebyshev (spatial) basis functions. A temporal feedback, i.e., auto-regressive, kernel was also used, which was not represented as a Volterra expansion, but instead, a simple vector of learned weights and delay. The output predicted by the model was the spike density of the CA3 PCs belonging to the neural mass

The CNMM adopts a filter or kernel-based architecture that uses Volterra expansions to represent the impulse response of the system as a linear weighted sum of nonlinear basis functions (Marmarelis, 1993). The structure of the model, shown in Fig. 3, was strongly influenced by the true anatomical circuits in the EC-DG-CA3 system which are the perforant path, the mossy fibers, and the CA3 associational connections. Each input population’s spike density was transformed via convolution with its corresponding feedforward kernel (EC, DG, and adjacent CA3 populations). Additionally, a temporal feedback kernel was used to capture the dynamics of the associational connections of the neural mass’s own CA3 PCs as well as the recurrent connections within CA3 from its basket cells (i.e., the CA3PC*→ *CA3BC*→ *CA3PC loop). Collectively, these convolutions produced four different time series (3 from the inputs, 1 from the feedback) which were weighted and summed to generate the model’s output prediction. For using the CNMM to predict CA3 spike density, we specify the model as SD-CNMM (to differentiate it from its use in LFP prediction).

The EC, DG, and feedback kernels are temporal kernels that only operate in the time domain, representing the impulse response of the system due to a single unit of spike density. The CA3 associational kernel (hereby referred to as the coupling kernel), is three-dimensional and operates in both the spatial and temporal domains. The three dimensions come from two dimensions of space (corresponding to the proximodistal and septotemporal axes) and one dimension of time. The spatial components of the kernel represent how the magnitude of neighboring neural mass activity is integrated as a function of distance (i.e., spatial lag) from the target neural mass. Spatio-temporal kernels (i.e., the coupling kernels) were constructed by taking the outer products of two spatial components and one temporal component. The feedforward kernels (EC, DG, and coupling) were composed of a linear weighted sum of basis functions and are equivalent to first-order Volterra kernels. In the temporal domain, Laguerre basis functions were used. The Laguerre polynomials have been demonstrated to be excellent candidate basis functions in representing physiological and neural dynamics (Hu et al., 2015; Marmarelis, 1993; Song et al., 2009). They exhibit orthogonality, good convergence, and significantly reduce the number of terms in Volterra models. The *n*th order discrete-time orthonormal Laguerre function is given by Eq. (5).

5 $$\begin{aligned} L_{n,\alpha }(\tau ) = {\left\{ \begin{array}{ll} (-1)^\tau \alpha ^{(n-\tau )/2}(1-\alpha )^{1/2}\times \sum _{k=0}^{\tau }{(-1)^k\left( {\begin{array}{c}\tau \\ k\end{array}}\right) \left( {\begin{array}{c}n\\ k\end{array}}\right) \alpha ^{\tau -k}(1-\alpha )^k}, & 0 \le \tau < n \\ (-1)^n\alpha ^{(\tau -n)/2}(1-\alpha )^{1/2}\times \sum _{k=0}^{n}{(-1)^k\left( {\begin{array}{c}\tau \\ k\end{array}}\right) \left( {\begin{array}{c}n\\ k\end{array}}\right) \alpha ^{n-k}(1-\alpha )^k},&n \le \tau \le M \end{array}\right. } \end{aligned}$$

The discrete-time Laguerre parameter *α * determines the rate of exponential decay and is defined on the interval *0< α < 1*. Note, the notation $$\left( {\begin{array}{c}n\\ k\end{array}}\right) $$ represents the combination function which computes the number of ways to choose *k* elements from a set of *n* elements without regard to order.

In the spatial domain, Chebyshev basis functions were used which exhibit orthogonality, symmetry, computational efficiency, fast convergence, and numerical stability. Past works have demonstrated the efficacy of Chebyshev polynomials in modeling discrete, spatially varying random fields and ophthalmic surfaces (Liu & Zhang, 2017; Rodrigues et al., 2019). The *n*th order Chebyshev polynomial is given by Eq. (6).

6 $$\begin{aligned} T_n(x) = \sum _{k=0}^{\lfloor n/2 \rfloor } \left( {\begin{array}{c}n\\ 2k\end{array}}\right) x^{n-2k} (x^2 - 1)^k, \quad x \in [-1, 1] \end{aligned}$$

The above equation defines the Chebyshev polynomials on the interval *x ∈ [-1, 1]*. The equation was modified to be scaled to an open parameter *r* as shown in Eq. (7).

7 $$\begin{aligned} T_{n,r}(x) = \sum _{k=0}^{\lfloor n/2 \rfloor } \left( {\begin{array}{c}n\\ 2k\end{array}}\right) \frac{x^{n-2k} (x^2 - r^2)^k}{r^n}, \quad x \in [-r, r] \end{aligned}$$

**Fig. 4:**
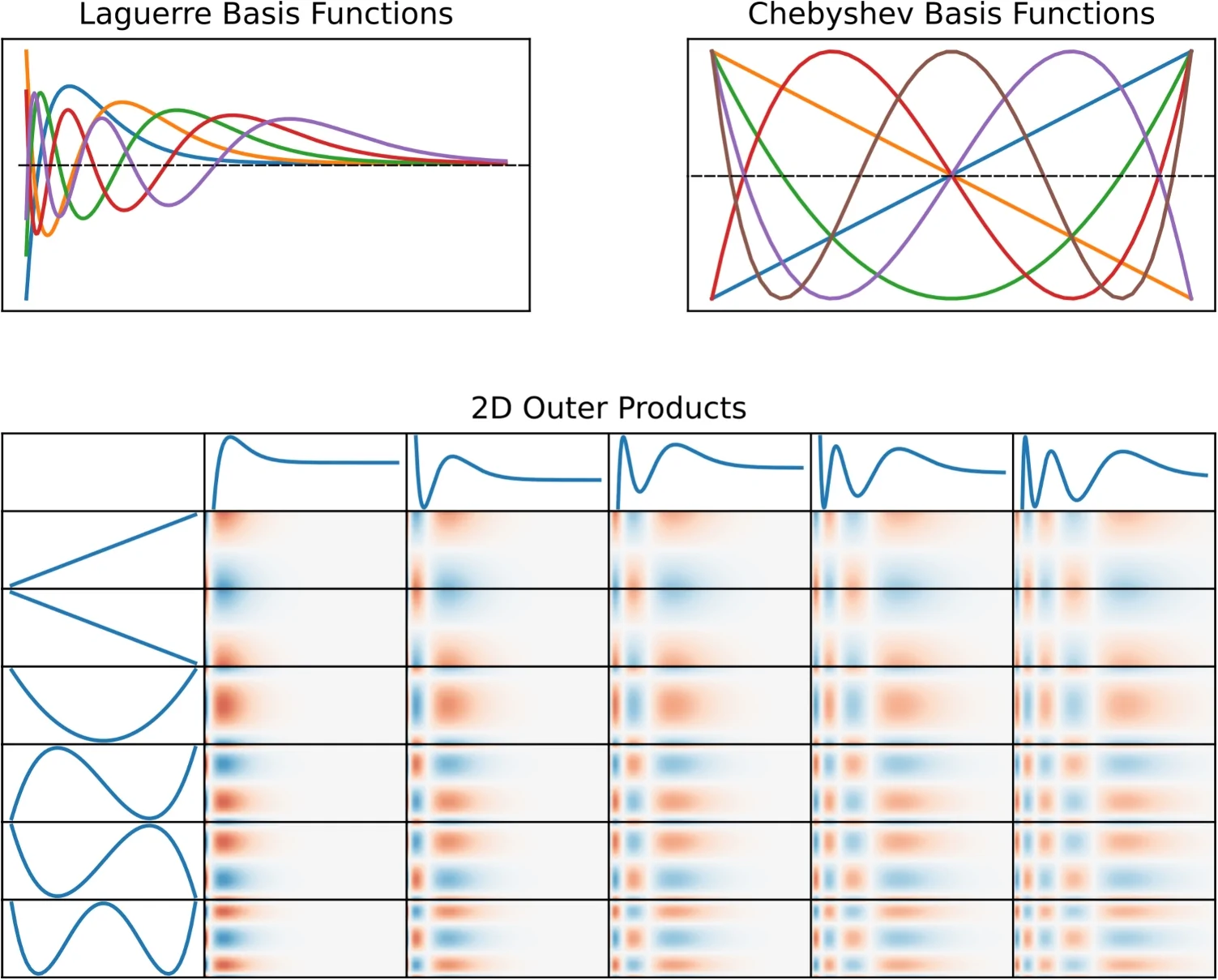
**Laguerre and Chebyshev basis functions.** The Laguerre (top-left) and Chebyshev (top-right) polynomials are shown. The outer products between these two families of polynomials (bottom panel) represent the bases for a hypothetical two-dimensional spatio-temporal kernel

For purposes of this study, the parameter *r* represents the maximal spatial lag (i.e., within the CA3) that the Chebyshev basis functions can span. Laguerre and Chebyshev basis functions, and their outer products, are visualized in Fig. 4.

As shown above, the basis functions give rise to the open and learnable metaparameters *r* and *α *. In the CNMM, each kernel has independent metaparameters that must be optimized to minimize prediction error. The EC and DG kernels have an *α * value, which dictates the decay rate of the Laguerre basis functions and determines the temporal dynamics of the kernel. The coupling kernel has an *α * value and two *r* values: $r_x$ (spatial extent in the proximodistal direction) and $r_y$ (spatial extent in the septotemporal direction). Both $r_x$ and $r_y$ dictate the spatial extent of the Chebyshev basis functions, determining how far in space (from the neural mass being modeled) the kernel recruits neighboring CA3 neural mass inputs from. The feedback kernel has a single metaparameter, $N_{delays}$, which determines the amount of initial zero-padding applied to the temporal filter. Collectively, these four kernels are governed by a total of six free metaparameters which must be optimized for output prediction of a single CNMM. Additional metaparameters to the model exist, but are set to a constant value and are not optimized during training. They are referred to as static metaparameters. The memory window *M* defines the kernel time length, determining the temporal extent to which past dynamics influence the present. The value of *M* is set to 1000 ms to ensure a sufficient duration is provided to capture all dynamics and transients without capturing false dynamics from events too far in the past. The static metaparameters $N_{t}$, $N_{x}$, and $N_{y}$ are the basis orders, which determine the maximum order of the basis functions in the temporal domain (Laguerre bases) and two spatial domains (Chebyshev bases), respectively. For example, if $N_{t} = 3$, then the corresponding kernel contains Laguerre basis functions up to third order. There also exist static metaparameters $P_{t}$, $P_{x}$, and $P_{y}$ which determine the power of each kernel in their respective domains. For example, $P_{t} = 3$ indicates that the temporal kernel will include basis function terms of up to the third power. Static metaparameter values for the CNMM are summarized in Table 1. As the value of the static metaparameters increase, so do the total number of weights in the model. Therefore, static metaparameters were tuned manually to provide a sufficiently rich basis set that could fit complex waveforms and produce an accurate model while also maintaining a feasible number of weights to keep computational demand low. The choice of static metaparameter values was guided by the trade-off between model accuracy and number of total weights in the model. We observed diminishing returns when increasing both the basis orders and the powers of the basis functions: incrementally increasing these values progressively resulted in smaller gains in accuracy but led to a rising increase in the number of terms, especially with the coupling kernels in which both spatial and temporal domains are represented. The final set of static metaparameters was therefore chosen to achieve a practical balance between model accuracy and computational tractability.

**Table 1:** Static metaparameters of the CNMM

	Kernel			
Static Metaparameter	EC	DG	Coupling	Feedback
$N_t$	8	8	3	–
$N_x$	–	–	4	–
$N_y$	–	–	4	–
$P_t$	5	5	3	–
$P_x$	–	–	3	–
$P_y$	–	–	3	–
*M*	1000 ms			

The following equations show how the CNMM computes the output, $\hat{v}^{(x_0,y_0)}(t)$, at a neural mass with center located at $(x_0, y_0)$:

8 $$\begin{aligned} \begin{aligned} \hat{v}^{(x_0,y_0)}(t) = \sum _{reg}^{\{EC,DG\}} \sum _{\tau = 0}^{M}{K_{reg}(\tau )u^{(x_0,y_0)}_{reg}(t-\tau )} + \sum _{\tau = 0}^{M}K_B(\tau )\hat{v}^{(x_0,y_0)}(t-\Delta t - \tau ) \\ + \sum _{i=-r_x}^{r_x}\sum _{j=-r_y}^{r_y}\sum _{\tau = 0}^{M}K_C(i,j,\tau )u_C(x_0-i,y_0-j,t-\tau ) \end{aligned} \end{aligned}$$

9 $$\begin{aligned} K_{reg}(\tau ) = \sum _{n_t=1}^{N_t} \sum _{p_t = 1}^{P_t} w_{reg}(n_t,p_t)(L_{n_t,\alpha } (\tau ))^{p_t}, \quad reg \in \{EC, DG\} \end{aligned}$$

10 $$\begin{aligned} \begin{aligned} K_C(i,j,\tau )= \sum _{n_x=1}^{N_x}\sum _{n_y=1}^{N_y}\sum _{n_t=1}^{N_t}\sum _{p_x=1}^{P_x}\sum _{p_y=1}^{P_y}\sum _{p_t=1}^{P_t} \\ w_C(n_t,p_t,n_x,p_x,n_y,p_y)(T_{n_x,r_x}(i))^{p_x}\otimes (T_{n_y,r_y}(j))^{p_y}\otimes (L_{n_t,\alpha }(\tau ))^{p_t} \end{aligned} \end{aligned}$$

where $\hat{v}^{(x_0,y_0)}(t)$ represents the model output which is either the CA3 spike density for SD-CNMM or the transmembrane current for LFP-CNMM (see Section 2.4 for details). The superscript $(x_0,y_0)$ denotes the location of the neural mass of interest. Eq. (8) is the main equation that relates the output $\hat{v}^{(x_0,y_0)}(t)$ from the inputs *u(t)*. The equation can be interpreted as the sum of all four kernel contributions. The first summation in Eq. (8) represents the contributions from the EC and DG kernels by computing the time-domain convolutions of the kernels $K_{EC}(\tau )$ and $K_{DG}(\tau )$ with their respective input signals $u_{EC}^{(x_0,y_0)}(t)$ and $u_{DG}^{(x_0,y_0)}(t)$. The second summation in Eq. (8) represents the contribution from the feedback kernel $K_B(\tau )$ and its convolution with the auto-regressive signal $\hat{v}^{(x_0,y_0)}(t-\Delta t)$ with *Δ t=2ms* (the sampling period). The feedback kernel $K_B(\tau )$ is a vector of freely learnable coefficients and governed by the learnable metaparameter $N_{delays}$ which determines the amount of initial zero-padding of the kernel. The third summation in Eq. (8) represents the contribution from the coupling kernel $K_C(i,j,\tau )$ which performs a three-dimensional convolution that results in a time-domain signal (with space held at a constant value). The signal $u_C(x,y,t)$ represents the CA3 spike density time series at neural masses located at *(x,y)*. Eq. (9) represents the general form of the temporal EC and DG kernels as a linear combination of different orders (up to $N_t$) and powers (up to $P_t$) of the Laguerre basis functions with weight coefficients $w_{EC}$ and $w_{DG}$ that were estimated during model optimization. Similarly, Eq. (10) represents the general form of the spatio-temporal coupling kernel as a linear combination of different orders and powers of the Chebyshev and Laguerre basis functions with weight coefficients $w_C$. Note, the *⊗ * symbol represents the outer product which expands the individual one-dimensional basis functions resulting in a three-dimensional kernel. Additionally, the learnable metaparameter *α * will have different values across the different kernels.

### CNMM as a forward model

To demonstrate the CNMM’s feasibility as a forward model for more directly observable macroscopic signals, we extended it to predict LFPs in CA3 (referred to as LFP-CNMM). Kernel-based methods for calculating LFPs have been explored by multiple previous studies. In Telenczuk et al. (2020), LFPs originating from hippocampal pyramidal cells were estimated via summation of unitary LFPs (uLFP) which were modeled by convolution of spike times and uLFP kernels. This work was expanded in Tesler et al. (2022) in which both LFP and MEG signals were computed via convolution of kernels with the mean firing rates of neuronal populations in a mean-field model (functionally equivalent to a NMM). In Ness et al. (2025), the authors performed analysis on the validity of LFP and EEG estimation from population firing rates and concluded that kernel-based methods were best suited for scenarios involving asymmetric neurons that receive spatially clustered and highly correlated synaptic inputs, such as in pyramidal cells. The authors also noted the importance of validating kernel-based methods against ground truth data coming from biophysical models since past works were often derived from networks of point-neurons, making it unclear how accurate their predictions were. This limitation was addressed in Hagen et al. (2022) in which spatiotemporal kernels were convolved with population spike rates to predict extracellular potentials and could accurately replicate ground truth signals derived from a biophysical model. We propose a similar kernel-based approach which was also validated using a biophysically detailed model of CA3.

**Fig. 5:**
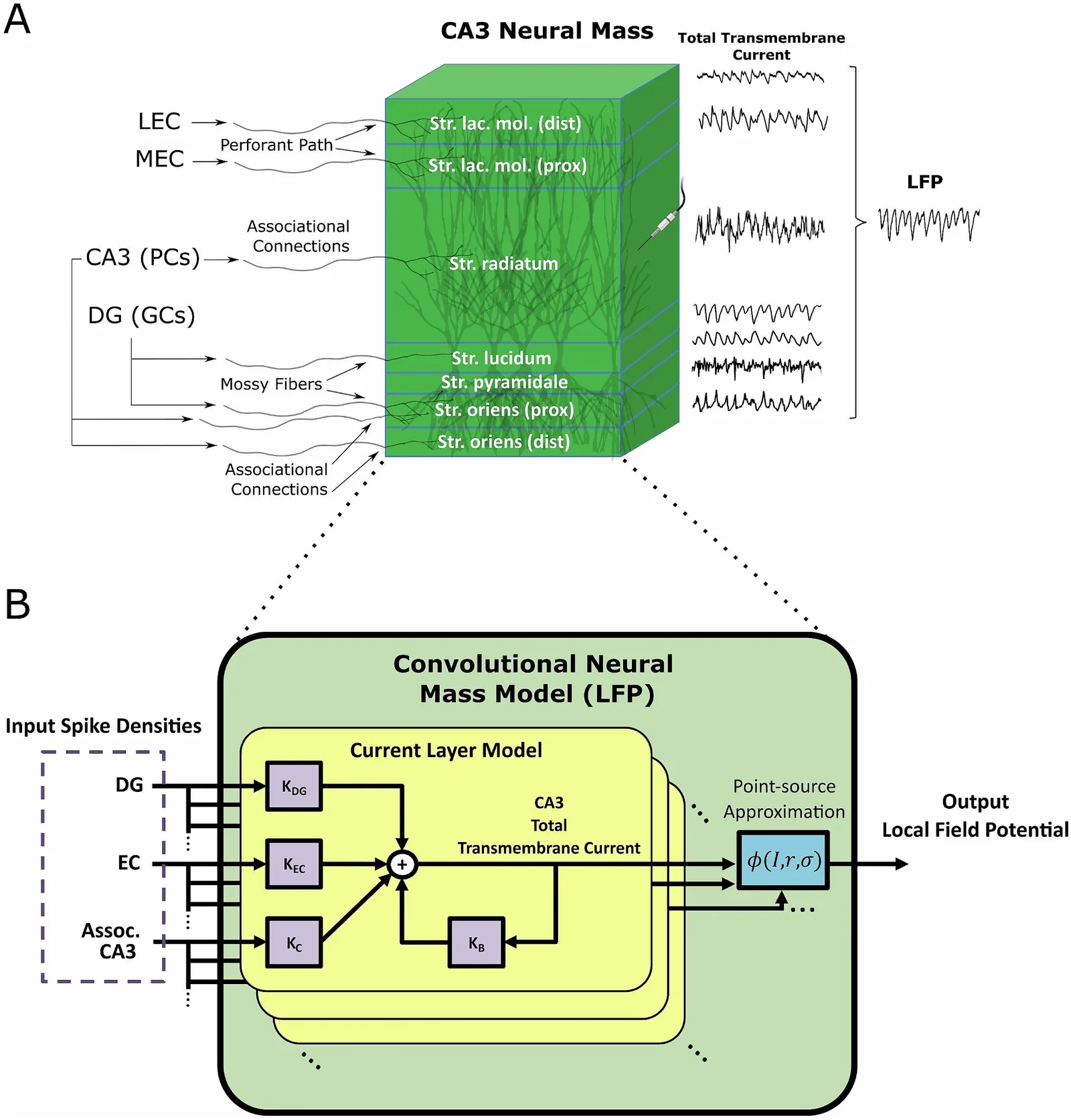
LFP-CNMM architecture. (**A**) A CA3 neural mass was divided into seven layers, each receiving synaptic inputs from specific subregions. The current layer model predicted total transmembrane currents at each layer and was used to calculate the local field potential (LFP). (**B**) The CNMM architecture was extended to predict LFP signals (LFP-CNMM). The depicted architecture is for a single CA3 neural mass. The model was composed of seven current layer models (in yellow), each used for prediction of the total transmembrane current of a specific layer in CA3. The total transmembrane currents were converted into an LFP prediction via the point-source approximation equation (depicted as blue box)

It has been well established that the major origin of extracellular fields and LFPs are transmembrane currents, especially those originating from synaptic activity (Buzsáki et al., 2012). Therefore, rather than directly predicting LFP, we developed layer-specific models that predict the total transmembrane current of a neural mass at different strata (layers) of CA3. These layer-specific models are referred to as current layer models. The layers of CA3 were vertically stacked along the radial axis within the LSM. We defined the total transmembrane current of a layer as the sum of all transmembrane currents from all compartments of pyramidal and basket cells (within the LSM) contained within the neural mass and layer. For this study, the layers included in the LFP-CNMM reflect those represented in the LSM which were: str. lacunosum moleculare (distal), str. lacunosum moleculare (proximal), str. radiatum, str. lucidum, str. pyramidale, str. oriens (proximal), and str. oriens (distal). Anatomically, the input projections to CA3 are organized in a layer-specific fashion. LEC and MEC inputs target the lacunosum moleculare layers, CA3 associational connections target the radiatum and oriens layers, and DG mossy fibers target the lucidum and proximal oriens layers as depicted in Fig. 5A. Figure 5B illustrates the adapted CNMM architecture for LFP prediction, called current layer models. A current layer model is defined as a model that represents an individual CA3 layer of a neural mass and predicts its total transmembrane current. It has an identical kernel-based architecture to the SD-CNMM with the exception that total transmembrane current was predicted rather than spike density. The inputs to a current layer model are the same as the inputs to the SD-CNMM: spike densities from DG, EC, and adjacent CA3 masses. Seven current layer models were generated, each representing a different stratum within the CA3 neural mass. The LFP-CNMM prediction for a single neural mass is a linear combination of the seven current layer models, resulting in a single output LFP signal. Total transmembrane currents predicted from the current layer models were assumed to be point sources and were converted into LFPs via the point-source approximation equation given by Eq. (11).

11 $$\begin{aligned} \phi =\sum _{k=1}^{N}{\frac{I_k}{4\pi \sigma r_k}} \end{aligned}$$

where *ϕ * represents the LFP, $I_k$ represents the current from the *k*th point source at a distance of $r_k$ from the recording site. *N* denotes the number of current sources (i.e., the number of layers: seven). Conductivity of the medium (i.e., extracellular space) is denoted by *σ * and was set to a constant value of 0.3 S/m. Because the total transmembrane current from a single layer was simplified to a single point, the value of $r_k$ was defined as the distance between the recording electrode and the center of the *k*th layer.

As with spike density prediction, the ground truth training data for LFPs came from the LSM simulation. The neuron models in the LSM spanned the seven layers of CA3. The layer-specific transmembrane currents of every cell were recorded. At each layer, the transmembrane currents of each cell were summed, resulting in the total transmembrane current of that layer. These total transmembrane currents served as the ground truth data that the current layer models (of the LFP-CNMM) aimed to predict. Additionally, ground truth LFP data was generated by using the point-source approximation in which every cell’s compartment was considered a current point source.

### Model optimization

As discussed earlier, the CNMM paradigm gives rise to a set of six open metaparameters, which must be optimized individually for each neural mass. Due to the 6-dimensional parameter space that must be searched, the particle swarm optimization (PSO) algorithm was chosen (Kennedy & Eberhart, 1995). Briefly, PSO is a population-based evolutionary algorithm in which multiple candidate solutions, or “particles”, are randomly initialized across the parameter space and iteratively updated to minimize the loss function. The algorithm takes advantage of exploration (sampling of diverse parameter combinations) and exploitation (convergence toward the best solution). With each iteration, every particle moves to a different position in the parameter space based on a stochastically weighted sum of the current global best solution, the particle’s personal best solution, and the particle’s previous velocity. The collective behavior of the particles leads to an efficient and effective optimization algorithm capable of navigating a complex parameter landscape. PSO contains three weights which define the contributions of the particle’s velocity (called inertia), the population’s global best solution (called the social weight), and the particle’s personal best solution (called the cognitive weight) in determining the particle’s next position. In this study, the PSO algorithm was implemented using the Inspyred library (Garrett, 2023) (in Python 3.8), which provides a framework for solving optimization problems using genetic and evolutionary algorithms. The LSM was used to simulate 40,000 ms of simulated data: 32,000 ms for training and 8,000 ms for validating the CNMM (i.e., an 80/20 train-validation split). 32 neural masses, that spanned the CA3, were optimized individually for spike density prediction resulting in a set of six optimized metaparameters and kernel weights for each individual neural mass. During both training and validation, the spike density inputs to the coupling kernel ($u_C(x,y,t)$) were taken directly from the ground-truth LSM simulations rather than from the outputs of other CNMM units. In other words, the CNMM was trained and evaluated in an open-loop (conditional) manner using the full set of LSM-derived inputs for every neural mass. The number of particles for PSO was set to 200. Optimization was run for 2 hours per neural mass, which resulted in approximately 140 generations and allowed convergence to be reached. The inertia, social weight, and cognitive weight were set to: 0.25, 1.0, and 2.0, respectively. Mean-squared error between CNMM prediction and LSM-derived ground-truth validation data was used as the main loss function. Additionally, estimation of all kernel weights was accomplished using ridge regression (Hoerl & Kennard, 1970) which provides regularization by penalizing large-magnitude coefficients and preventing overfitting while maintaining model stability. Ridge regression has an open regularization parameter $\lambda _{ridge}$ that determines the strength of penalizing coefficients and was set to a value of 10. For LFP prediction, optimization was performed on a single CA3 neural mass that was closest to the center of the CA3 region. This representative location was chosen to demonstrate the CNMM’s capability as a forward model for LFP signals while maintaining computational tractability across the full population of neural masses. The seven current layer models of the LFP-CNMM were each optimized individually using the previously described process. Additionally, the ground truth data were LSM-derived transmembrane currents rather than spike density. Optimization was performed on the University of Southern California’s Center for Advanced Research Computing (CARC) nodes using AMD EPYC 7513 CPUs.

Optimization was performed independently for each neural mass due to computational constraints, as jointly optimizing all 32 neural masses simultaneously would have required searching a much higher-dimensional parameter space. Because the CNMM receives ground-truth inputs from the LSM during both training and validation, this per-mass approach is consistent with the model’s design as a conditional input-output emulator. A limitation of this choice is that coupling kernels are not optimized with mutual consistency across neural masses, which would only be possible under a joint optimization of a closed-loop system.

## Results

### Accuracy in spike density prediction

**Fig. 6:**
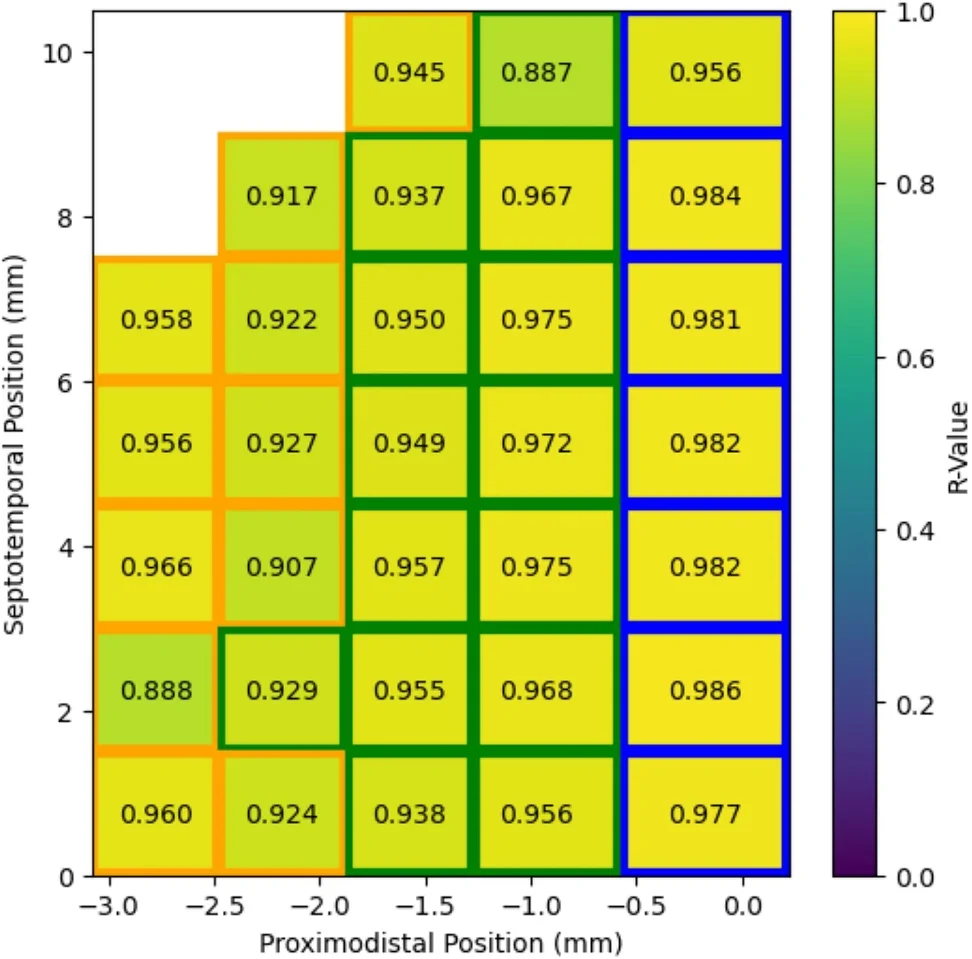
**CNMM spike density prediction accuracy.** A two-dimensional heatmap of *R*-values across 32 CA3 neural masses are shown over the septotemporal-proximodistal plane. Border colors of blue, green, and orange represent neural masses belonging to the CA3a, CA3b, and CA3c subfields, respectively

**Fig. 7:**
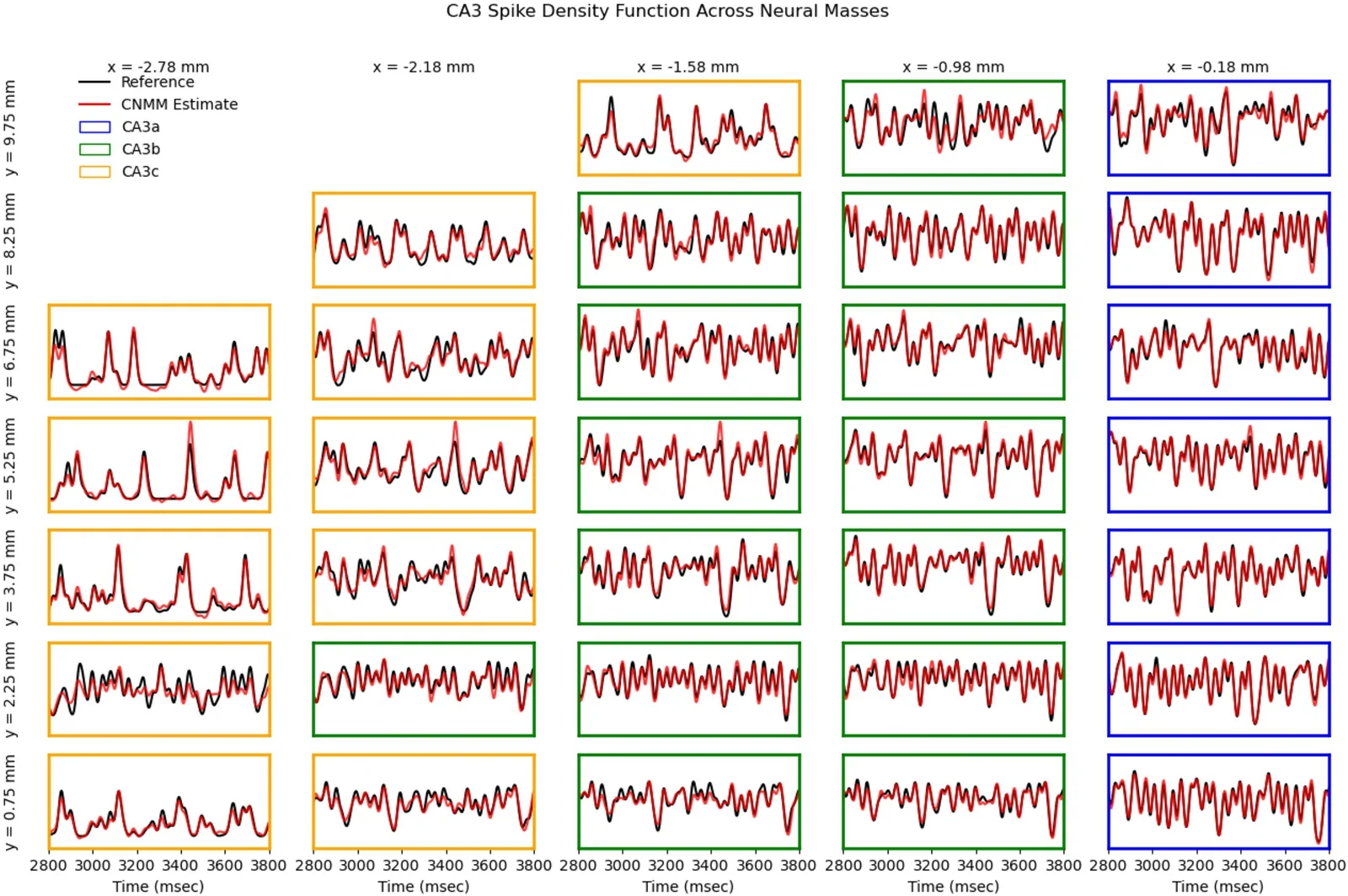
**CNMM spike density estimates.** Excerpts of 1,000 ms of CNMM spike density signal estimates (red traces) are superimposed onto the reference CA3 spike density signals (black traces). Traces are normalized and are therefore shown in arbitrary units. Each box represents a different neural mass. Border colors of blue, green, and orange represent neural masses belonging to the CA3a, CA3b, and CA3c subfields, respectively. The x and y values that span the top row and left column denote the centers of the neural masses within the CA3 topography along the proximodistal and septotemporal axes

**Fig. 8:**
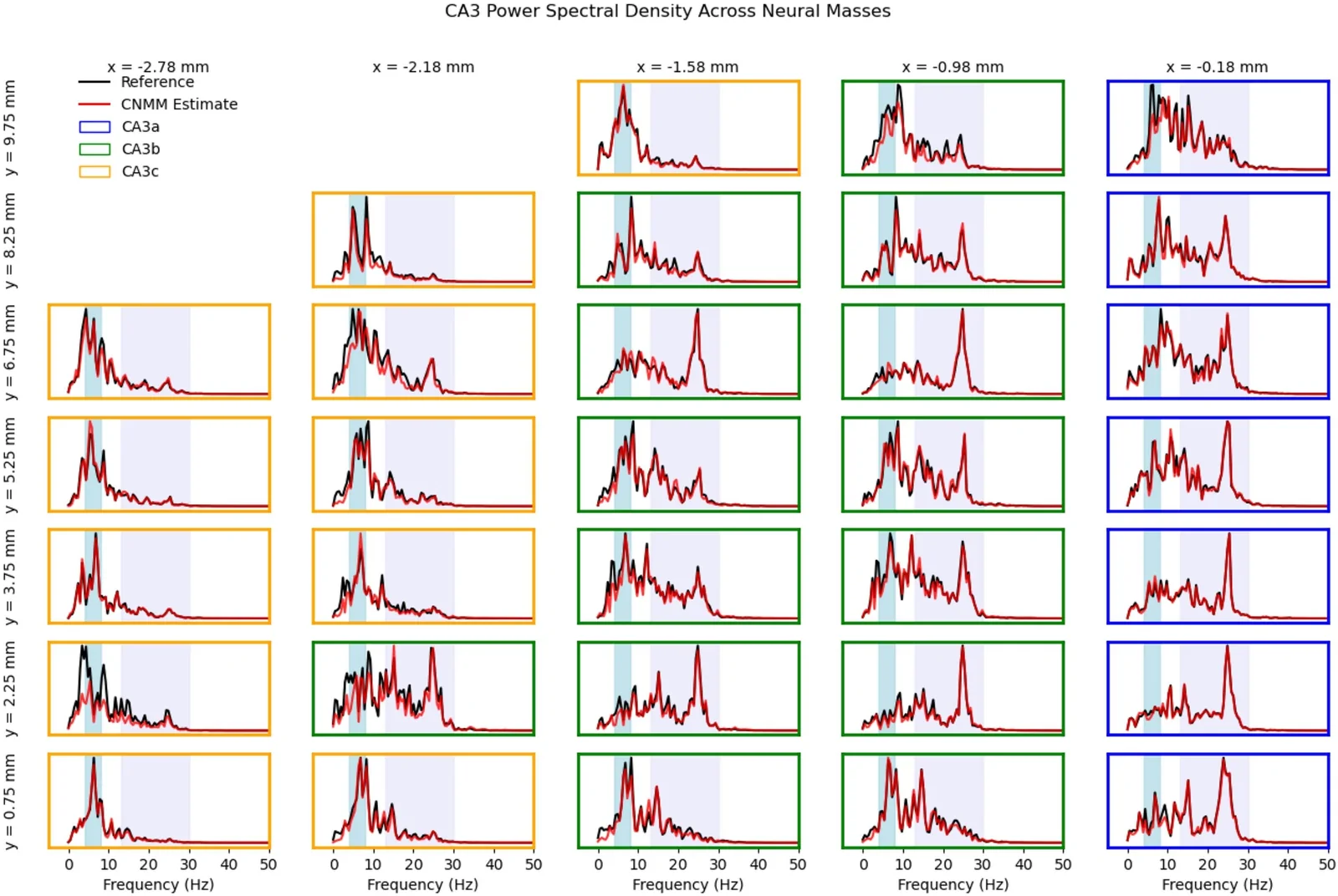
**CNMM power spectra.** Power spectra of the CNMM spike density signal estimates (red traces) are superimposed onto the power spectra of the reference signals (black traces). The vertical axis is shown in arbitrary units. The theta band (4-8 Hz) is represented by the light blue regions while the beta band (13-30 Hz) is shown in light purple

The CNMM’s accuracy was evaluated by measuring the correlation coefficient *R* between 8,000 ms of the reference CA3 spike density signal (i.e., the validation set from the simulated rat exploration task) and the CNMM’s estimated spike density signal. Accuracy was evaluated across the 32 optimized neural masses that spanned the extent of the CA3. The dimensions of the neural mass spatial bins were 0.2 mm (proximodistal) by 0.5 mm (septotemporal). All signals had a sampling period of 2 ms. Figure 6 shows the achieved *R* values across the neural masses. The results show the strong predictive power of the CNMM with minimum, maximum, and mean *R*-values of 0.887, 0.986, and 0.951 ± 0.027, respectively. Figure 7 visually compares the CNMM spike density estimates to the reference signals. In order to visualize the frequencies captured by the CNMM, the power spectral density (PSD) was computed (via Welch’s method) on the CNMM spike density estimates and reference signals shown in Fig. 8. The power spectra show that the CNMM is able to replicate oscillations in the frequencies exhibited by the LSM. Additionally, they show that CA3 mesoscopic dynamics from the CNMM and LSM exhibit oscillations predominantly in the theta band (4-8 Hz), and with increasing power in the high beta band (20-30 Hz) when approaching the distal end of CA3. The theta rhythm is known to be a hallmark of hippocampal activity (Nuñez & Buño, 2021) while the beta rhythm has been experimentally shown to be expressed by mice exploring novel environments (Berke et al., 2008). The increased power in the high beta band toward the distal end of CA3 is a consequence of the higher proportion of bursting-type CA3 pyramidal cells found in CA3a (Masukawa et al., 1982) and the increased recurrence of the associational connections in CA3a (Le Duigou et al., 2014) which elevate firing rates. Both of these gradients are expressed in the LSM. Collectively, these results demonstrate that the CNMM methodology is able to accurately replicate the mesoscale dynamics of the biophysically detailed mechanistic LSM across a range of frequencies. Moreover, the frequency content of the signals agree with experimental findings with regard to the behavior being modeled (exploration of a rat) as both models express oscillations in the theta and beta bands which are involved in exploration and locomotion.

To evaluate the CNMM’s ability to generalize over different inputs (i.e., tasks beyond rat exploration), a separate LSM simulation was executed such that all input MEC spiking activity was changed to a random homogeneous Poisson process with mean firing rate of 5.50 Hz. All other parameters of the LSM remained the same as described previously. Then, new ground truth spike density data was generated from this LSM simulation. The previously trained CNMM (i.e., metaparameters and weights optimized to the original rat exploration data) was used to estimate output spike density when given random MEC spiking input over the 32 neural masses shown in Fig. A2. Visually, sparse and lower frequency output activity was more difficult for the model to capture. Accuracy of these estimates were quantified by measuring the *R* values of the CNMM’s output to the ground truth at each neural mass shown in Fig. A3A3. In general, accuracy was still high, but with slight decreases in *R* value when compared to Fig. 6. Collectively, these results demonstrate that the CNMM was robust to a change in input statistics: from structured grid cell activity to random homogeneous Poisson spiking. However, this test was performed within the same LSM configuration with all other parameters the same as before. Broader generalization across different behavioral tasks, network states, or perturbations to synaptic parameters was not evaluated and remains an important direction for future work.

The values of the optimized metaparameters over the 32 neural masses are reported in Fig. A4. An example of the evolution of the metaparameter space during PSO is shown in Fig. A5 which visually demonstrates the convergence of each of the metaparameters. Additionally, the sensitivity of the CNMM can be inspected in Fig. A5 by looking at the mean squared error (i.e., the color of each dot) associated with each metaparameter value. Upon inspection, the accuracy of the CNMM was most sensitive to the *α * value for the DG kernel, the *r* values for the coupling kernel, and the $N_{delays}$ value of the feedback kernel as those metaparameters converged to a tight region, and values outside that region tended to produce higher prediction error.

### Kernel contributions in spike density estimation

**Fig. 9:**
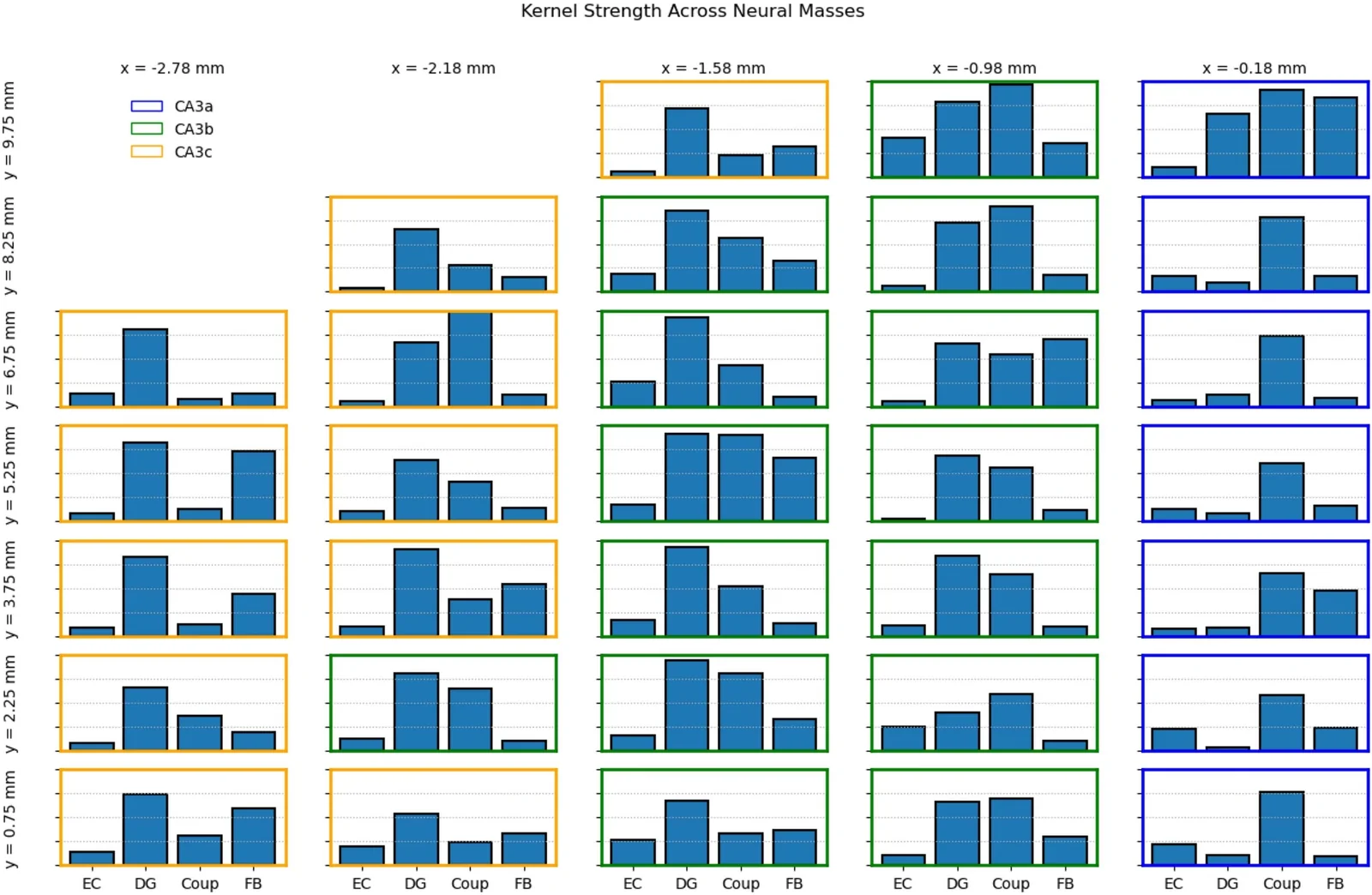
**CNMM kernel strengths.** Kernel strengths of the four kernels are shown across 32 CA3 neural masses. The vertical axis is in arbitrary units, but constant across all plots, allowing for direct comparison of kernel strengths between neural masses. Kernel strength of DG predominates on the proximal side of CA3 (CA3c) and decreases toward the distal side (CA3a). Meanwhile, coupling kernels have low strength on the proximal side and predominate on the distal side

One feature of the CNMM structure is the ability to quantify the relative contributions of different afferents to a population of neurons (i.e., neural mass). One way to accomplish this is by computing the strength of the optimized kernels using the root mean square (RMS) of the coefficients of the kernels shown in Eq. (12).

12 $$\begin{aligned} \rho = \sqrt{\frac{1}{N_{terms}}\sum _{i=1}^{N_{terms}}{k_i^2}} \end{aligned}$$

where *ρ * represents the kernel strength, $k_i$ represents the *i*th term (or coefficient) in the kernel, and $N_{terms}$ represents the number of terms in the kernel. This metric provides a method of quantifying the impact that each input (EC, DG, or associational connections) has on the target population (CA3). Moreover, kernel strengths can be compared across neural masses to characterize the differences in afferent integration as a function of space (i.e., position in the CA3 topography). Therefore, kernel strengths were computed for each kernel across all 32 CA3 neural masses, which were optimized for predicting spike density, and are shown in Fig. 9. Strength of the DG kernel predominates in the proximal side of CA3 and is significantly decreased at the distal side of CA3. On the other hand, coupling kernels predominate in the distal side and have low kernel strength in the proximal side. This suggests that the afferents from DG contribute most to the CA3 PC spiking in CA3c while associational connections contribute most in CA3a. The gradient of DG kernel strengths across the proximodistal axis can be explained by the fact that density of mossy fiber synapses are greatest in CA3c and lowest in CA3a which has been reflected in the LSM (Acsády et al., 1998; Yu et al., 2020). Additionally, CA3 associational connections have been shown to be less dense in CA3c and most dense in CA3a, which was expressed in the LSM and explains the kernel strength gradient for the coupling kernels (Ishizuka et al., 1990; Yu et al., 2020). Furthermore, the low coupling kernel strengths observed at the proximal boundary of CA3 (leftmost column of Fig. 9) may reflect an edge effect since there are fewer neighboring CA3 neural masses to recruit from.

**Fig. 10:**
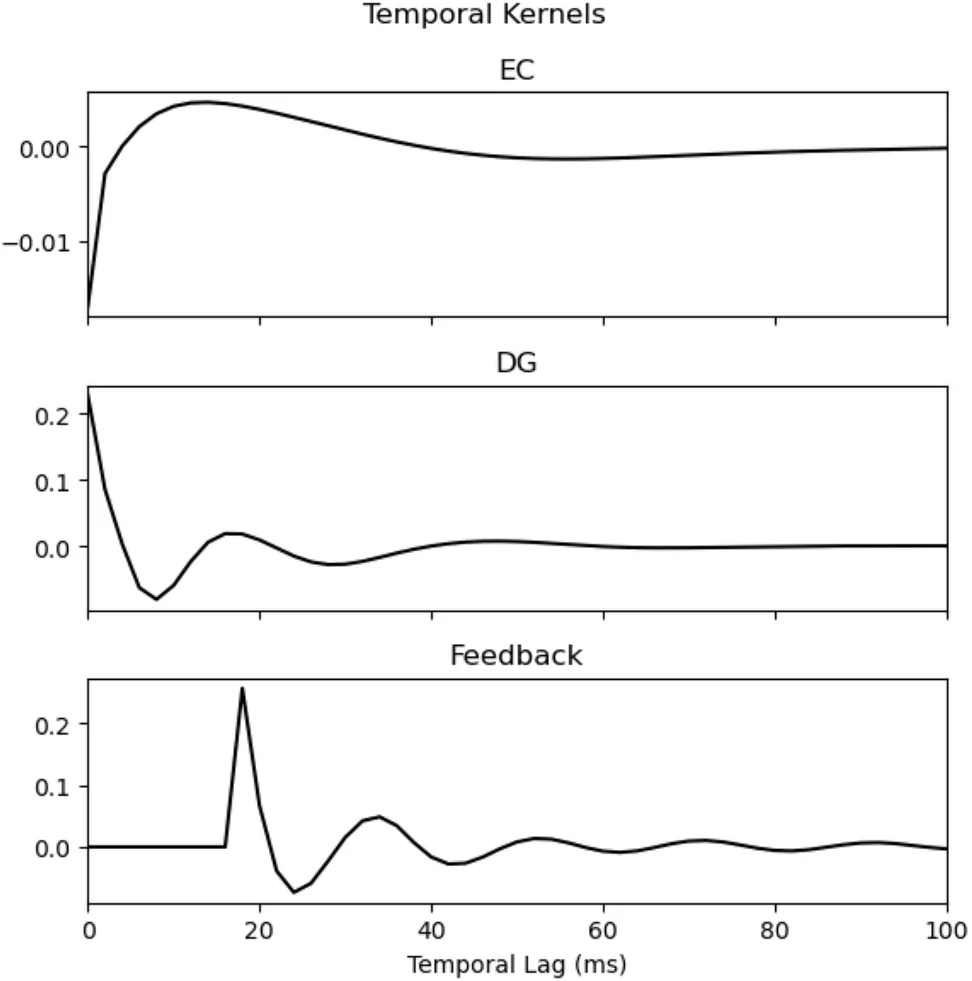
Temporal kernels from a single neural mass. The EC, DG, and feedback temporal kernels from a sample neural mass are shown. EC and DG kernels were derived from first-order Laguerre-Volterra expanded kernels while the feedback kernel was a simple vector of learnable coefficients with a learned amount of initial delay (i.e., zero-padding)

**Fig. 11:**
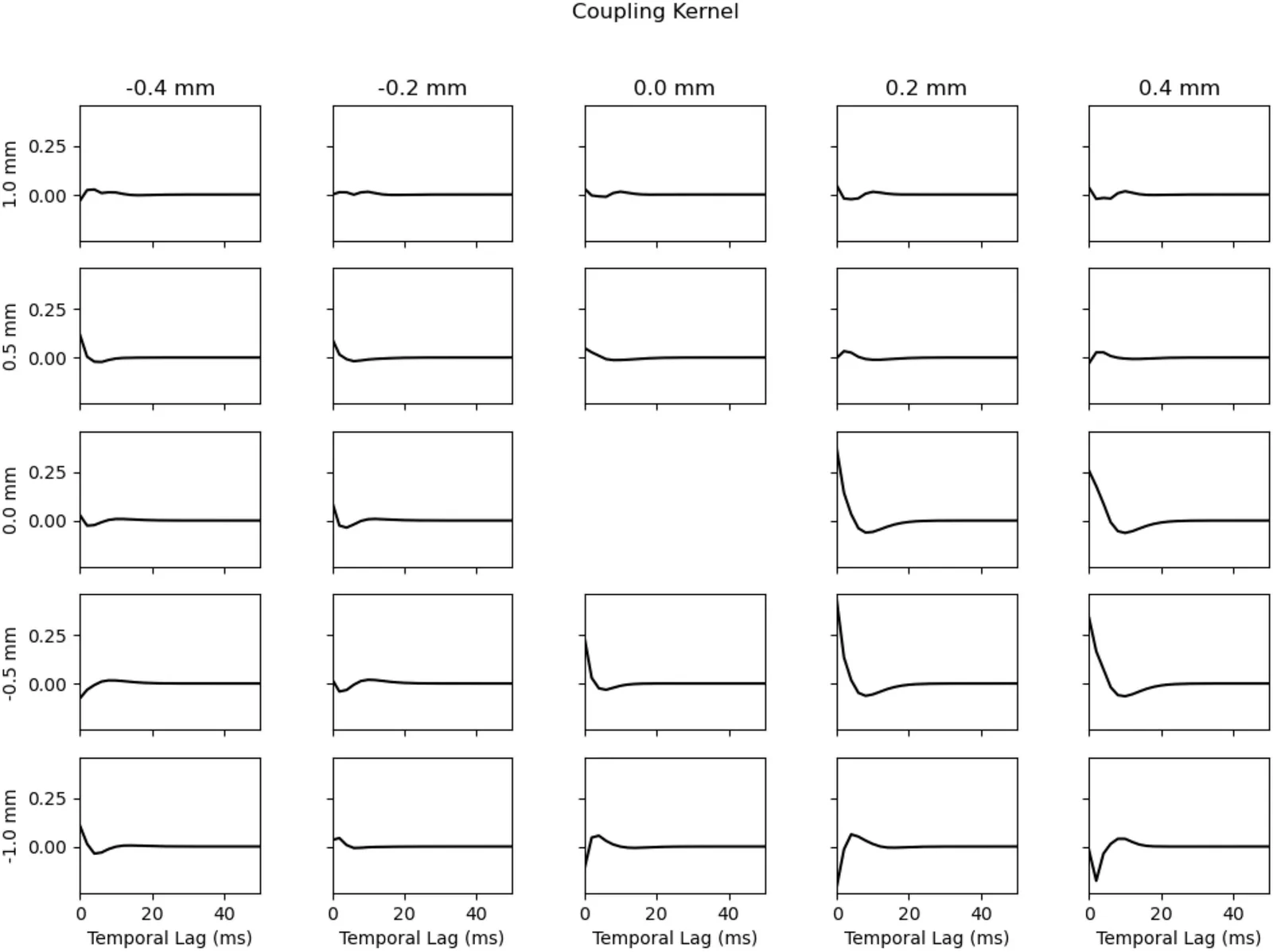
Coupling kernel from a single neural mass. A portion of the spatiotemporal coupling kernel is visualized as a 2D matrix of temporal kernels where each plot represents a different spatial lag with respect to the modeled neural mass. The coupling kernel exhibits various temporal profiles at different spatial lags that are mostly biphasic. Filters closer to the center and in the distal region have larger magnitudes. Proximodistal lags are printed in the top row while septotemporal lags are printed along the left column. The y-axis scaling is the same for all plots and is unitless

Sample representative kernels from a single neural mass, with center located at a proximodistal location of -0.98 mm and a septotemporal location of 6.75 mm, are shown in Fig. 10 (temporal kernels) and Fig. 11 (coupling kernel). The EC kernel is characterized by an initial inhibitory (negative) component followed by a relatively slow and low magnitude excitatory (positive) component. The relatively low strength of the EC kernel may be physiologically explained by the fact that EC afferents to CA3 PCs terminate in the most distal locations of the dendrites in stratum lacunosum moleculare, leading to significant attenuation in the resulting EPSP at the soma. Both DG and feedback kernels exhibit dampened oscillations, alternating between excitatory and inhibitory components. This behavior is consistent with the fact that both corresponding pathways strongly engage the recurrent excitatory-inhibitory (E-I) circuitry of CA3. Mossy fiber inputs provide sparse, yet powerful, excitation onto both CA3 pyramidal cells and basket cells, thereby driving strong feedforward inhibition in addition to direct excitation. Similarly, the associational pathway provides recurrent excitation among CA3 pyramidal cells while also engaging feedback inhibition through the PC-BC-PC loop. It is important to note that because the CNMM is an input-output model in which inhibitory interneurons are represented only implicitly, the negative components of the kernels should be interpreted as reflecting net suppressive effects in the transformation from inputs to outputs, rather than direct synaptic inhibition from specific interneuron populations. The feedback kernel was optimized to have an initial delay (zero-padding) of 18 ms. The coupling kernel exhibits different temporal profiles at different spatial lags that are mostly biphasic and are either initially inhibitory or excitatory.

### Accuracy in LFP prediction

**Fig. 12:**
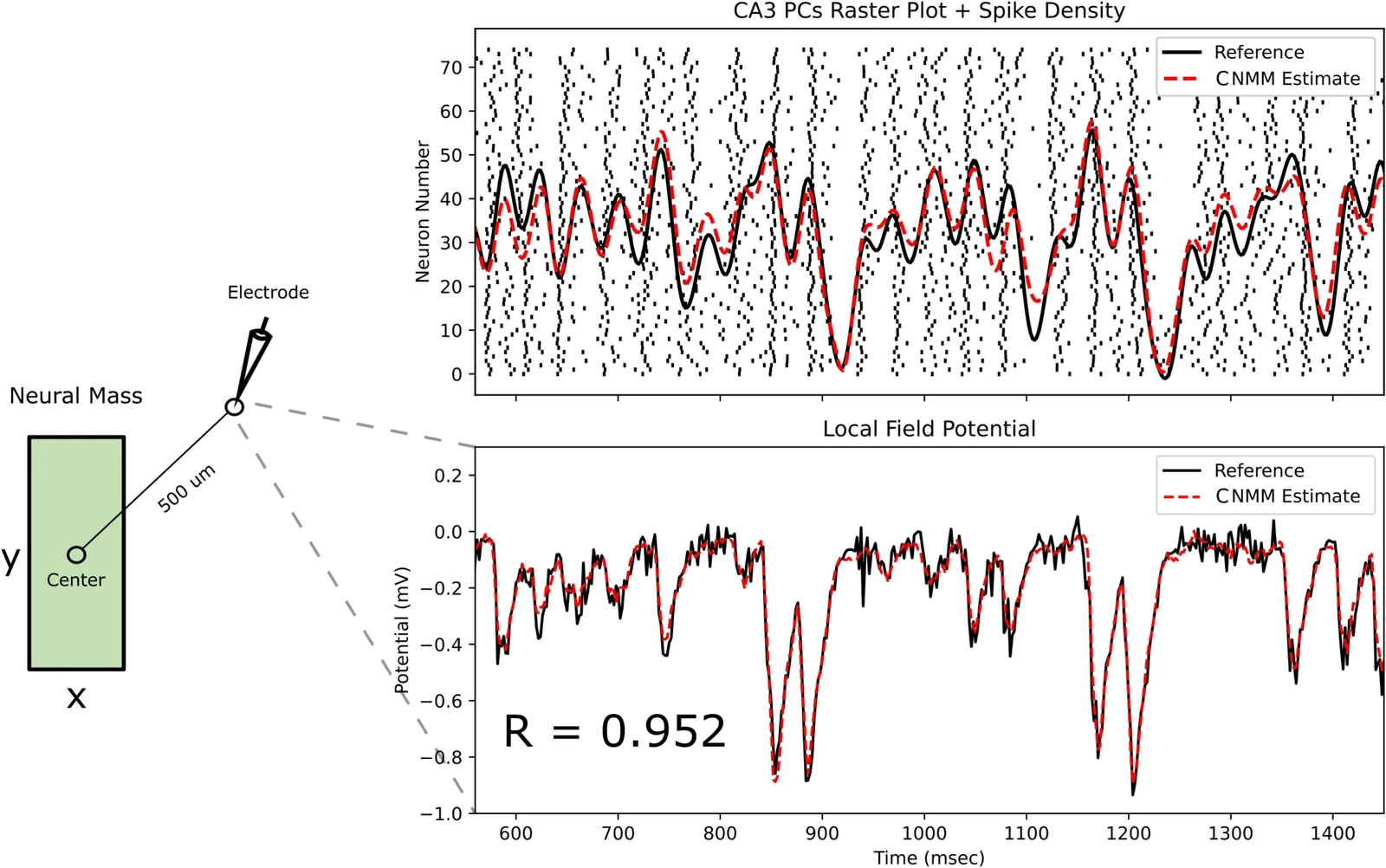
**CNMM LFP prediction.** (Left) A neural mass is viewed from a top-down perspective in the septotemporal-proximodistal plane with a single virtual electrode placed 500 *μ *m away from the center of the neural mass for LFP estimation. LFPs were computed using the point-source approximation for both the CNMM and LSM with respect to the position of the electrode. (Right) The top panel shows the raster plot of CA3 PCs (from LSM simulation) with corresponding reference (black) and CNMM estimate (red) spike density traces superimposed. The bottom panel shows the resulting LFPs from both reference and CNMM estimate superimposed. An *R*-value of 0.952 was achieved by the CNMM estimate

The accuracy of the extended CNMM for LFP prediction was assessed by computing the correlation coefficient between 8,000 ms of the CNMM predicted LFP and LSM derived LFP (i.e., the validation set from the simulated rat exploration task). A single CA3 neural mass, closest to the center of the CA3, was evaluated. In both models, a single virtual recording electrode was placed at a radial level matching the center of the stratum pyramidale layer and 500 *μ *m away from the center of the neural mass in the septal and distal (45$^{\circ }$ between septal and distal axes) directions as shown in the left portion of Fig. 12. Raster plots of CA3 PCs spiking within the neural mass from the LSM simulation are shown with superimposed reference and CNMM estimate spike density traces in the top plot of Fig. 12. The bottom plot shows the corresponding LFP signals from both LSM reference and CNMM estimate, computed using the point-source approximation with respect to the position of the virtual electrode. The CNMM achieved an *R*-value of 0.952 in LFP prediction for this single neural mass and electrode configuration. This result demonstrates the feasibility of extending the CNMM to forward modeling of LFPs.

### Computational efficiency

**Fig. 13:**
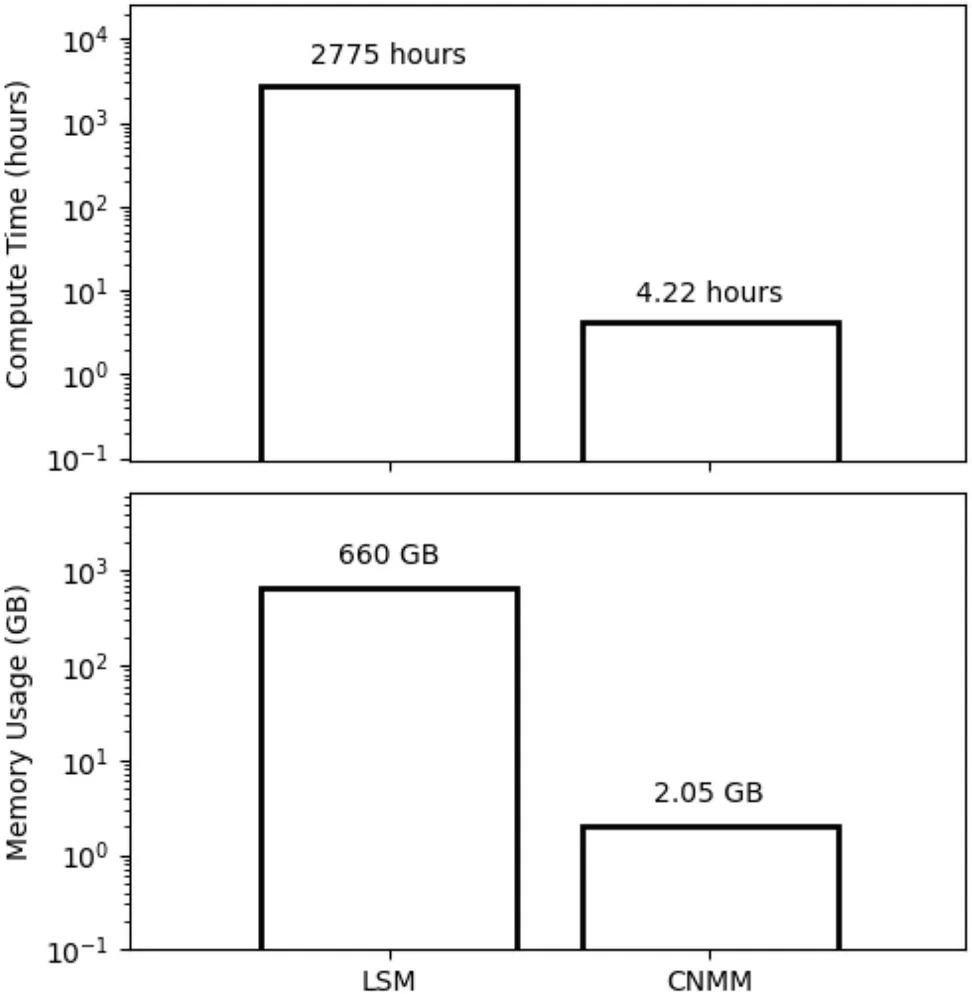
Computational efficiency comparison. Compute time and memory usage are compared between LSM and CNMM in simulations of 40,000 ms in duration. Y-axis is in log-scale. CNMM metrics were corrected by multiplying by the number of CA3 neural masses (304) contained in the model. CNMM achieves a 658-fold simulation speedup and a 322-fold reduction in memory usage

Computational efficiency of the CNMM was compared against the LSM by measuring compute time and memory usage for 40,000 ms of simulated time, shown in Fig. 13. Simulations were performed on the University of Southern California’s Center for Advanced Research Computing (CARC) nodes using AMD EPYC 7513 CPUs. LSM simulations used 100 cores in parallel. Four trials of LSM simulations were run. Compute times for LSM were calculated by multiplying the average wall-time (27.75 hours) and multiplying it by 100 resulting in 2,775 compute hours. Compute times for CNMM were estimated by recording the inference time for spike density prediction for 32 neural masses and multiplying the average run time by 304 (the number of total neural masses in the network) resulting in 4.22 hours of compute time. This linear extrapolation assumes that per-mass runtime remains relatively consistent across the CA3 extent. This calculation does not include the compute time required for optimizing the CNMM. The average memory usage in LSM simulations (4 trials) was 660.14 GB. The average memory usage for CNMM was calculated by multiplying the average memory usage of a single neural mass prediction by 304 resulting in a total of 2.05 GB. The CNMM achieves significant reduction in computational demand with a 658-fold simulation speedup and a 322-fold reduction in memory usage, allowing large-scale mesoscopic dynamics to be simulated on a single machine.

To provide a comprehensive assessment of computational requirements, Table 2 summarizes the costs associated with the complete SD-CNMM workflow, including LSM simulation, spike-density preprocessing, CNMM optimization, and trained-model inference. Notably, CNMM optimization using PSO represents a major component of the total pipeline cost. The optimization protocol used for the primary results in this study (referred to as “CNMM Optimization (Long)" in Table 2) required approximately 131,733 compute hours to optimize all 304 neural masses. To evaluate a more computationally efficient alternative, we also tested a reduced optimization protocol (referred to as “CNMM Optimization (Short)" in Table 2) in which the number of CPU cores was reduced from 200 to 20 and the optimization time per neural mass was shortened from 2 hours to 15 minutes. This reduced the total computational cost to 1,888 compute hours while maintaining a high mean correlation coefficient of *R = 0.944 ± 0.030* across the 32 neural masses.

**Table 2:** Computational costs for SD-CNMM workflow

Component	Compute Time (hours)^b^	Memory Usage	Cores Used
LSM Simulation	2,775	660 GB	100
Spike-Density Preprocessing	0.86	1.17 GB	1
CNMM Optimization (Long)^a^	131,733	185 GB	200
CNMM Optimization (Short)^a^	1,888	17.7 GB	20
Trained CNMM Inference^a^	4.22	2.05 GB	1

^a^Estimated by linear extrapolation from the 32 optimized neural masses to all 304 neural masses

^b^Compute time is reported in core-hours (wall-clock time multiplied by the number of cores used)

**Fig. 14:**
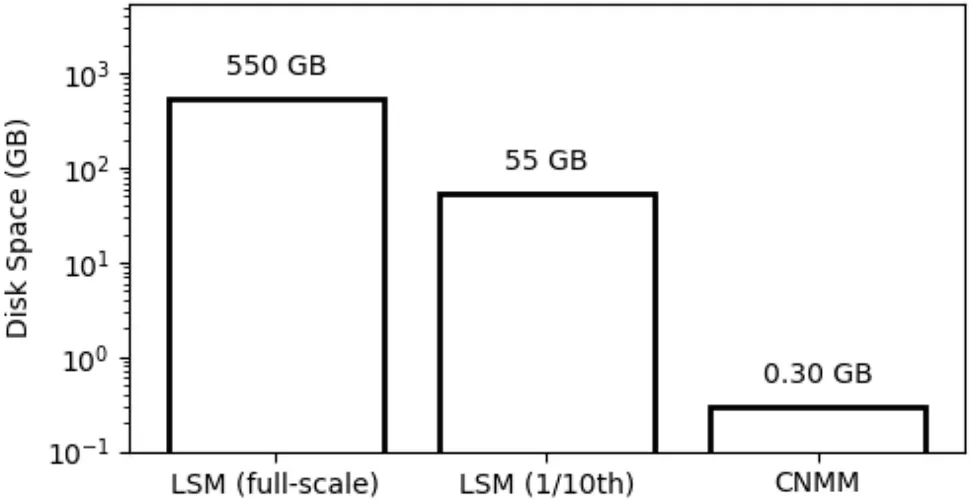
**Disk space requirements for LFP prediction.** Disk space requirements for LFP predictions are compared between LSM (full-scale and 1/10th scale) and CNMM. For LSM, disk space was found by measuring the size of the file containing all recorded transmembrane currents. For CNMM, disk space was found by measuring the file containing all spike densities. The disk space requirement for full-scale LSM LFP prediction was inferred because the file size increases proportionally to the number of neurons in the network. The file size requirement for CNMM is independent of the number of neurons in the network

The CNMM methodology offers even further reduction in computational demand in LFP prediction when compared to the LSM. In order to calculate LFP using the LSM, the transmembrane currents of every neuron must be recorded. This means that both the number of calculations (i.e., point-source approximations) and the number of recorded signals (i.e., disk space usage) will be proportional to the number of cells contained in a network. In this study, 1/10th the number of neurons of a true rat hippocampus were simulated and recorded, leading to a file size of 55 GB of recorded transmembrane currents. A simulation at full-scale would require approximately 550 GB of disk space for recorded transmembrane currents and 10 times more point-source approximations to obtain an LFP signal. On the other hand, the file size and number of calculations for the CNMM to predict LFP are independent of the number of neurons in the network because the model only requires input spike density signals for predictions. The disk space for storing spike density signals does not change with number of neurons in the network. In this study, the file containing all spike density signals consumed 0.30 GB of disk space resulting in a 183-fold reduction or 1833-fold reduction in disk space requirements when compared to LSM at 1/10th scale or at full-scale, respectively. Disk space requirements for LFP estimation are compared in Fig. 14.

## Discussion

Computational models have emerged as indispensable tools for understanding the complex interactions of the brain at various scales, offering valuable insights into its intricate mechanisms and functions. Detailed mechanistic multi-scale models enable the simulation of micro-scale interactions and their emergence as macro-scale activity, but simulation feasibility diminishes as the number of neurons modeled approaches that of a real brain and computational demands rise. For analysis at the meso-scale, this computational burden is largely alleviated using the neural mass modeling approach which significantly reduces the dimensionality of the model. Computational demand can be further reduced using input-output modeling techniques such as the Laguerre-Volterra framework. In this report, a novel spatially discretized convolutional neural mass model was proposed, taking aspects from both Laguerre-Volterra and neural mass models, offering a computationally efficient methodology for studying the rat hippocampus at the meso-scale.

The CNMM deviates from the traditional neural mass modeling framework in that population activity is modeled using a kernel-based input-output architecture rather than a system of coupled differential equations of inhibitory and excitatory populations. The CNMM approach mostly resembles that of a generalized linear model (GLM), such as the one from Pillow et al. (2008), in which spiking is estimated for individual retinal ganglion cells using a series of spatio-temporal and temporal kernels. However, unlike such GLMs, the CNMM utilizes the known organization of the hippocampus by creating input-specific kernels (e.g., for perforant path, mossy fibers, and associational connections), thereby increasing the mechanistic insight it provides into topographic transformations. One limitation of traditional NMMs is that they are often validated on highly simplified networks of identical leaky-integrate-and-fire (LIF) neurons with random connectivity and no regard for topographical or spatial aspects. This weakness is addressed in this study as the CNMM is trained and validated against an anatomically constrained, biophysically detailed mechanistic multi- and large-scale model (LSM) of the rat EC-DG-CA3 system under the behaviorally relevant conditions of random exploration. Moreover, the entire subregion being modeled (i.e., CA3) is discretized over its anatomical extent, adding a spatial component that traditional NMMs do not consider and enabling analysis of the system within the spatial domain.

The CNMM can be situated within a growing landscape of biologically grounded model reductions and coarse-graining in computational neuroscience. Several efforts have sought to derive efficient mesoscopic descriptions from detailed spiking networks while preserving biological interpretability. For example, Chang et al. (2025) recently introduced a Markovian framework to reduce homogeneous spiking networks to systems of ordinary differential equations capable of capturing firing rates, metastability, and bifurcation structure in finite-size networks. In Xiao et al. (2024), a coarse-graining framework was proposed that exploits local anatomical similarity across cortical regions by precomputing local population responses, achieving significant computational savings while retaining biological fidelity. Another approach has extended the mean-field modeling paradigm to capture mesoscopic activity from networks expressing dynamic synapses that exhibit short-term plasticity (STP) (Schmutz et al., 2020). This framework was further extended at the hippocampus by Pietras et al. (2022) in which the authors developed a stochastic mesoscopic model that captures metastable dynamics and hippocampal replay. Also within the hippocampus, Tesler et al. (2024) constructed a bottom-up mean-field model that could replicate multiple brain oscillations in macro-scale simulations of CA1 with morpho-anatomical connectivity from a large network of extended generalized integrate-and-fire (EGLIF) neurons. While these approaches generally derive reduced dynamical equations from assumptions of spike statistics or network structure, the CNMM takes a complementary, data-driven approach by learning effective spatio-temporal input-output transformations (kernels) directly from data generated by a large-scale mechanistic model. This kernel-based structure, which is informed by the anatomical organization of the target subregion (i.e., CA3), offers significant computational savings during inference and the ability to analyze how different afferent pathways are integrated across space—features that are not directly accessible in most analytically derived mean-field reductions.

We have demonstrated that the CNMM can accurately predict CA3 PC spike density functions (SD-CNMM) across multiple neural masses that span the CA3 (Fig. 7) as well as LFP (LFP-CNMM) at a single neural mass (Fig. 12). Furthermore, the CNMM was able to capture the frequency ranges expressed in spike density signals (Fig. 8) which included oscillations in the theta band and increased power in the high beta band in neural masses located toward the distal end of CA3, consistent with experimental results and known topographical CA3 gradients. The CNMM also showed robustness when tested with a different, random homogeneous Poisson spiking input from MEC (instead of structured grid cell activity) by maintaining high accuracy across the 32 neural masses (Fig. A2 and Fig. A3) using parameters obtained from optimizing to the original rat exploration task. This result demonstrates generalization to an altered input regime. In addition to accurate predictions, the CNMM offers significant computational advantages over the LSM once trained. When comparing inference (i.e., simulation after optimization), the CNMM achieved a 658-fold reduction in compute time and a 322-fold reduction in memory usage relative to the LSM (simulating 1/10th the number of neurons of a full-scale hippocampus). For LFP prediction, the CNMM also reduced disk space usage by a factor of 183 compared to the LSM. It should be noted that these gains in efficiency refer specifically to the cost of running the CNMM post-training and do not include the upfront costs of preprocessing spike-density data or CNMM optimization via PSO. As shown in Table 2, optimization for all 304 neural masses represents a significant cost under the protocol used in this study. However, we also demonstrated that this cost can be substantially reduced by lowering the number of particles used in PSO and the amount of optimization time dedicated to each neural mass while maintaining similar predictive accuracy (*R = 0.944 ± 0.030*). Compute times and memory usage for the full set of 304 neural masses were estimated by linear extrapolation from measurements on the 32 optimized neural masses. We expect that kernel metaparameters could potentially be interpolated across space rather than requiring independent optimization for every neural mass which would drastically decrease computational cost of optimization and increase scalability, but a future study would be needed to verify this. Even larger reduction factors are expected when the LSM is simulated at full-scale since LSM runtimes and memory consumption scale with number of neurons modeled while CNMM computational demands remain relatively constant. This reduced computational burden allows for simulation of meso-scale neuronal dynamics to be performed with higher accessibility and without the need for high performance computing clusters. Due to its increased efficiency, simulations of much longer timescales (minutes to hours) are made possible.

The low computational cost of the CNMM for predicting LFPs also makes it a promising candidate for forward modeling within broader multi-scale simulation frameworks. While the current implementation was only evaluated on a single neural mass and electrode position, the results establish the feasibility of employing CNMM for accurate LFP prediction. For example in Tesler et al. (2022), a mean-field model was developed for LFP and MEG prediction within the Virtual Brain (Sanzleon et al., 2013), resulting in a computationally efficient forward model. In the same spirit, the CNMM can also be applied as an efficient forward modeling tool in large-scale network simulations, bridging the gap between the micro- and macro-scales. This study demonstrated the feasibility of forward modeling through one of many observable modalities—the local field potential. The LFP is one of the most important signals because of its high temporal resolution and its integration of local networks. This combination allows for LFPs to detect brain oscillations (or “rhythms") which play a major role in determining brain states and identifying neuropathologies. This is especially true in the hippocampus, where brain rhythms are known to be altered under pathological conditions such as depression, schizophrenia, epilepsy, and Alzheimer’s disease (Fernández-Ruiz, 2016). There is also evidence to suggest that LFP recordings in the hippocampus exhibit a degree of spatial discreteness, with characteristic lengths comparable to the dimensions of the neural masses defined in this study, indicating that the spatial resolution of LFPs aligns with our model (Benito et al., 2014). However, the CNMM approach can potentially apply to other neuroimaging signals such as functional ultrasound (fUS) which is often assumed to be linearly related to neuronal activity via the hemodynamic response function (Montaldo et al., 2022). The fUS paradigm entails three-dimensional voxels with spatial resolutions of about 100 *μ *m, which are highly comparable to the neural mass spatial bins defined in our study, making fUS a strong alternative neuroimaging signal for CNMM forward modeling. The prediction of multiple observable signals from a single framework enables a multi-modal analysis of co-registered signals that originate from the same neural activity. Integration of these modalities leverages the complementary strengths of each signal. For example, EEG offers high temporal resolution but poor spatial resolution, while fMRI provides the opposite. Fusion of these modalities can compensate for individual limitations, thus revealing dynamic brain activity not visible in isolation and allowing for a more holistic view on neural function (Sui et al., 2012). Lastly, multi-modal forward modeling also provides multiple points of validation to experimental results, ultimately leading to model improvements.

The kernels that compose the CNMM provide another feature in enabling direct quantification of how a neural population differentially integrates its afferents across topographical locations. In CA3, it was shown that the distribution of kernel strengths significantly varied depending on the proximodistal location of the neural mass: at the proximal end (toward CA3c), afferents from DG predominated over other synaptic inputs while at the distal end (toward CA3a), afferents from associational CA3 connections predominated (Fig. 9). While the LSM contains detailed anatomical and biophysical parameters, extracting the net effective weighting of different afferents on CA3 population output is not straightforward, as it requires disentangling many interacting factors (e.g., synaptic densities, synaptic weights, and neuronal biophysics). In contrast, the CNMM kernel strength directly measures the effective impulse response of each neural mass and collapses these underlying biological factors into a single, quantitative metric. This provides a more direct and interpretable way to compare how CA3 populations differentially weigh their inputs across space. In this study, physiological correlates were identified, providing a biological explanation to the differences in kernel strengths across the CA3 topography. These include topographical gradients in the density of mossy fiber inputs and size of associational connection fields within CA3 (Acsády et al., 1998; Ishizuka et al., 1990). Although the CNMM, due to its input-output nature, provides less mechanistic insight than the LSM, it still offers more biological interpretability than other abstract methodologies (such as artificial neural networks) as its architecture was influenced by the true anatomical connections in the EC-DG-CA3 system and transforms spike density signals originating from the actual afferents of CA3. The abstract kernels that compose the CNMM allow for the analysis and characterization of the complex input-output transformations that CA3 performs on its inputs. This study did not include an in-depth analysis on the extracted kernels of the CNMM. However, a potential study may reveal specific “kernel signatures" that may provide clinical insight. For example, perturbations to the hippocampal LSM may involve altering properties such as neuron excitability, synaptic weights, synaptic time constants, connectivity, biophysics, etc., to mimic a potential drug effect or pathology. Analysis of the kernels extracted from a perturbed neural system may reveal changes to the temporal or spatial profile of the kernels (i.e., the kernel signatures) when compared to the kernels extracted from the baseline simulation. To put it concisely, kernel signatures are the observable manifestation of mechanism(s) reflected at the meso-scale and captured by the profile of the kernels. These kernel signatures could potentially serve as markers for certain neurological conditions. Future studies extracting kernel signatures from experimental or patient data would be needed to evaluate whether they can aid in diagnosing such conditions. This concept has been hinted at in the Laguerre-Volterra literature. In Kang et al. (2010), kernels were extracted from *in vitro* CA3 PCs in both control and epileptogenic conditions. They found that first order kernels derived from epileptogenic neurons expressed a wider peak followed by multiple ripples in comparison to control neurons. Another study developed a Laguerre-Volterra network model for predicting spike times from LFPs in the subthalamic nucleus (STN) of patients with Parkinson’s Disease (PD) (Kostoglou et al., 2024). The authors identified three functional clusters of data recordings, each with their own distinct kernel signatures, and found that areas exhibiting characteristics of one cluster were associated with the greatest improvement in alleviating PD symptoms following deep brain stimulation. Therefore, kernel signatures may provide insight in identifying the optimal target for deep brain stimulation in patients with PD. These examples highlight a potential future application for the CNMM approach: extracting kernels and identification of kernel signatures may reveal mechanistic insight into the state of the brain using only observable input-output data (i.e., neuroimaging signals).

Although the CNMM succeeds in producing accurate meso-scale dynamics while minimizing computational load, there are important tradeoffs and limitations in the methodology that should be noted. First, the CNMM loses direct mechanistic control and insight into the system that the LSM provides. As an input-output model, the CNMM does not simulate the underlying biology, but instead, aims to make direct mathematical mappings from inputs to outputs. However, as discussed earlier, not all mechanistic insight is lost in the CNMM; the architecture of the model allows for the analysis of how a neural population integrates its different inputs while the extraction of kernels and kernel signatures may provide mechanistic insight through analysis of observable signals. Another limitation is the loss of granularity at the individual neuron level. As a neural mass model, the CNMM intrinsically represents the meso-scale and trades information encoded at the neuron level for the population level. However, evidence supports that information encoded at the population level is synergistic with information encoded at the cellular level (Hsieh et al., 2019). One methodological limitation of this study is that the simulated rat trajectory used to drive grid cell MEC input in the LSM to generate the training and validation data were produced using a simplified stochastic model in which speed, direction, and movement duration were sampled from uniform distributions with reflective boundary conditions that do not fully reflect the statistical structure of real rodent movement. Future work could incorporate recorded trajectories from actual rodents to generate more realistic input patterns for the LSM and CNMM. Another limitation in this study is that model performance was evaluated on a validation set, which was also used to guide metaparameter selection during PSO. While kernel weights were estimated exclusively from the training set via ridge regression, the validation set influenced the search over metaparameters. This train-validation protocol, though not ideal, was adopted due to the high computational cost of generating data from the large-scale mechanistic model. Furthermore, this study did not assess variability due to the stochastic nature of PSO. Future work could address sensitivity of the model (e.g., accuracy and kernels) to different initializations and randomness. Another limitation of the CNMM’s current implementation is that it operates in an open-loop (conditional) manner such that spike density inputs to the coupling kernels come directly from ground truth data (derived from the LSM) rather than in a closed-loop recursive manner by other CNMM units. This is a consequence of optimizing on the subset of 32 non-adjacent neural masses rather than the full set of 304. Therefore, the CNMM should currently be viewed as a biologically structured input-output reduction that can predict the activity of individual neural masses conditioned on afferents from neighboring CA3 activity. Development of a closed-loop version in which multiple CNMM units drive one another would be a valuable direction for future work. Additionally, while the CNMM demonstrated robustness to random MEC inputs, further generalization across different behavioral tasks, network perturbations, or different modeling assumptions was not assessed and should be explored in future studies. A further limitation stems from the implicit representation of inhibition. Inhibitory basket cells are explicitly simulated in the LSM but are not modeled as a distinct input population in the CNMM. Instead, their contributions are absorbed within the learned kernels. While the CNMM can reproduce net inhibitory effects on CA3 population activity, it does not enable separate analysis or perturbation of inhibitory mechanisms. This may constrain the model’s interpretability and generalization in contexts where inhibition is altered or the target of study, such as under pathological conditions or pharmacological interventions targeting GABAergic transmission. In its current form, the CNMM relies heavily on the LSM for generating input-output data for training and validation. This means that for every perturbation or condition that the CNMM aims to represent, an accompanying LSM simulation must be run. This reliance on the LSM also highlights another limitation of the CNMM in that the extracted input-output relationships reflect that of the LSM rather than the true biological system (the hippocampus). Though the LSM is a biophysically detailed model, agreement between the CNMM and the LSM should not be interpreted as capturing true hippocampal dynamics. Rather, the CNMM is capable of reproducing meso-scale dynamics generated by the LSM under specific test conditions. Further validation against experimental recordings would be required in order to show the CNMM can truly capture hippocampal activity. Furthermore, the main signal used in this study was the spike density function, which is a signal that estimates the firing rate of neurons over time and was derived by post-processing all spike trains in the LSM. Moreover, the workflow presented here assumed knowledge of the exact connectivity map of input neurons to output CA3 pyramidal cells leading to the generation of the exact input spike densities that a neural mass receives. In reality, this exact connectivity map cannot be known. At the same time, the spike density signal is not directly observable in experimental settings. Instead, this signal is inferred using analytical methods such as kernel smoothing of peristimulus time histograms (PSTH) in protocols that use multi-electrode arrays or high-density probes (Cunningham et al., 2010). As capabilities of brain-machine interfaces continue to advance rapidly, determination of spike density is likely to become increasingly common in experimental research and applications—making this approach even more directly relevant. To further extend the utility of the CNMM to clinical applications, its paradigm may be extended to input-output data that are experimentally observable. Several practical challenges must be addressed to achieve this. First, the size of neural masses would need to be defined based on the spatial coverage and resolution of the available recording technology, rather than using idealized spatial bins. Second, the input subregions (EC and DG) would also need to be subdivided into their own input neural masses. In this extended paradigm, input signals would be recorded from their subregion of origin. This extension would require both EC and DG kernels to include spatial components (in addition to their temporal components) because afferents of CA3 would originate from multiple spatially distributed input neural masses. Third, high-quality simultaneous recordings from both input and output subregions would be required so that population-level signals can be used directly for training. As discussed earlier, LFP and fUS constitute excellent candidates as macroscopic neural signals for the CNMM because of their spatio-temporal resolutions and direct observability. Fourth, the exact connectivity map from input neurons to each CA3 neural mass is unknown in real tissue; the model would therefore need to either incorporate anatomical priors or learn effective connectivity during optimization. Finally, validating the learned kernels would be more challenging without access to ground-truth data from a detailed model such as the LSM; approaches such as cross-validation across sessions or animals would likely be necessary. Future efforts to extend the CNMM paradigm for analyzing experimental data will substantially enhance its utility, enabling iterative comparisons of empirically derived and simulation-based kernels through the “virtuous loop"—ultimately refining the model and augmenting its predictive power. Additionally, this extension would enable the input-output characterization of the true biological system rather than a model of it (i.e., LSM).

While foundation models trained on large neural datasets are becoming increasingly powerful, the CNMM offers distinct advantages that remain valuable even in an era of purely data-driven approaches. Unlike foundation models, which often function as black boxes, the CNMM provides interpretable kernels that directly estimate the effective impulse responses and allow quantitative analysis of how a neural population integrates its different inputs. By grounding its architecture in known anatomical connectivity and enabling the extraction of kernel signatures, the CNMM also provides a framework for examining meso-scale neural processes that is more directly interpretable than large foundation models. This interpretability, combined with its relatively low data requirements and computational efficiency, makes the CNMM suitable for scenarios where large-scale training data are limited or where mechanistic insight is prioritized over raw predictive power.

This pilot study has laid the foundation for a novel computational methodology that utilizes aspects of input-output modeling, neural mass modeling, and forward modeling. We have demonstrated that the CNMM framework is successful at accurately predicting meso-scale dynamics across the CA3. It offers a promising foundation for forward modeling that could support meso-scale analysis of neural systems across their spatial extent, while greatly reducing the computational demands typically associated with detailed large-scale mechanistic models. Notably, while the CNMM can replicate the population dynamics observed in the LSM using a fraction of the computational resources, the objective of this work is not to replace the LSM with the CNMM. Instead, both models play essential, complementary and synergistic roles, working together to strengthen each other’s relevance and capabilities. The biological detail that the LSM provides enables scientists to tune virtually any mechanism and observe its effect at multiple scales. The CNMM can then be used to extract kernels from these LSM simulations and potentially identify kernel signatures that are tied to that mechanism. Kernel extraction from empirical data could then be used to help characterize the underlying neural condition of the subject based on its similarity to identified kernel signatures, or to validate that the simulation-derived kernel signatures match empirically-derived ones, therefore improving model validity. Such applications highlight the potential for kernel-based analyses to bridge computational predictions with real-world observations and support future efforts toward more precise diagnostics and targeted research strategies. Ultimately, by integrating detailed mechanistic simulations with efficient meso-scale modeling, the CNMM paradigm offers a scalable framework for advancing our understanding of hippocampal function and providing a potential foundation for future studies of dysfunction in both research and clinical contexts.

## Supplementary Information

Below is the link to the electronic supplementary material.

Supplementary file 1 (pdf 1020 KB)

## Data Availability

Example Python code is provided, which includes the class implementation of the CNMM, optimization data of a sample neural mass (including optimized metaparameters, weights, and kernels), input-output data, and a script that plots the results of a single optimized neural mass with its prediction of spike density. Code is available in our public Github repository at: https://github.com/NESCOM-Lab/CNMM.

## Code Availability

Example Python code is provided, which includes the class implementation of the CNMM, optimization data of a sample neural mass (including optimized metaparameters, weights, and kernels), input-output data, and a script that plots the results of a single optimized neural mass with its prediction of spike density. Code is available in our public Github repository at: https://github.com/NESCOM-Lab/CNMM.
